# A Flexible Synthesis of ^68^Ga-Labeled Carbonic Anhydrase IX (CAIX)-Targeted Molecules via CBT/1,2-Aminothiol Click Reaction

**DOI:** 10.3390/molecules24010023

**Published:** 2018-12-21

**Authors:** Kuo-Ting Chen, Kevin Nguyen, Christian Ieritano, Feng Gao, Yann Seimbille

**Affiliations:** 1 Department of Radiology and Nuclear Medicine, Erasmus MC, University Medical Center Rotterdam, Wytemaweg 80, 3015 CN Rotterdam, The Netherlands; k.chen@erasmusmc.nl; 2 Life Sciences Division, TRIUMF, 4004 Wesbrook Mall, Vancouver, BC V6T2A3, Canada; knguyen7@ualberta.ca (K.N.); cieritano@edu.uwaterloo.ca (C.I.); rggaofeng@hotmail.com (F.G.)

**Keywords:** carbonic anhydrase IX, ^68^Ga-labeling, click reaction, compound library

## Abstract

We herein describe a flexible synthesis of a small library of ^68^Ga-labeled CAIX-targeted molecules via an orthogonal 2-cyanobenzothiazole (CBT)/1,2-aminothiol click reaction. Three novel CBT-functionalized chelators (**1**–**3**) were successfully synthesized and labeled with the positron emitter gallium-68. Cross-ligation between the pre-labeled bifunctional chelators (BFCs) and the 1,2-aminothiol-acetazolamide derivatives (**8** and **9**) yielded six new ^68^Ga-labeled CAIX ligands with high radiochemical yields. The click reaction conditions were optimized to improve the reaction rate for applications with short half-life radionuclides. Overall, our methodology allows for a simple and efficient radiosynthetic route to produce a variety of ^68^Ga-labeled imaging agents for tumor hypoxia.

## 1. Introduction

Positron tomography scanners detect pairs of γ-rays originating from a radionuclide decaying by positron emission. The signal recorded by the scanner allows in vivo visualization and quantification of biological processes at the cellular or molecular level. Due to its high sensitivity and non-invasive properties, positron emission tomography (PET) plays an important role in cancer patient management, as a diagnostic and prognostic tool. ^68^Ga is one of the most attractive positron emitters for PET imaging due to its physical properties, such as short half-life (t_1/2_ = 67.7 min) and high positron abundance (β^+^: 89%), and its availability via a ^68^Ge/^68^Ga generator. The success of the [^68^Ga]DOTATE (NETSPOT^®^) in the PET imaging of somatostatin receptor–positive neuroendocrine tumors (NETs), as well as recent the U.S. Food and Drug Administration (FDA) approval of this agent, demonstrate the growing interest in the utility of ^68^Ga in PET imaging [1]. Moreover, its coordination chemistry and chemical tools are continuously being improved, facilitating the development of novel PET tracers based on this radiometal [2,3,4].

Tumor hypoxia represents a negative therapeutic indicator due to its multiple contributions to tumor invasiveness, radiotherapy and chemotherapy resistances [5,6]. Therefore, targeted imaging of hypoxic tumors would allow for the assessment of the response to antineoplastic treatments. Carbonic anhydrase IX (CAIX) is a transmembrane metalloenzyme that is strongly upregulated by hypoxia-inducible factor 1-α (HIF1-α) under tumor hypoxia [7]. The function of CAIX is to maintain intracellular acid-base homeostasis by catalyzing the interconversion of carbon dioxide (CO_2_) and bicarbonate (HCO_3_^−^); thus CAIX assists malignant cancer cell survival in the absence of oxygen [8,9]. Overexpression of CAIX has been reported in many types of malignancies, such as renal cell carcinoma, bladder, breast, lung and ovarian cancers. However, its expression in normal tissues is highly restricted [10,11,12,13,14]. Moreover, unlike the other members of this enzyme family, CAIX is abundantly present on the extracellular membrane of cancer cells. Therefore, CAIX represents a promising biomarker for tumor hypoxia detection.

**Figure 1:**
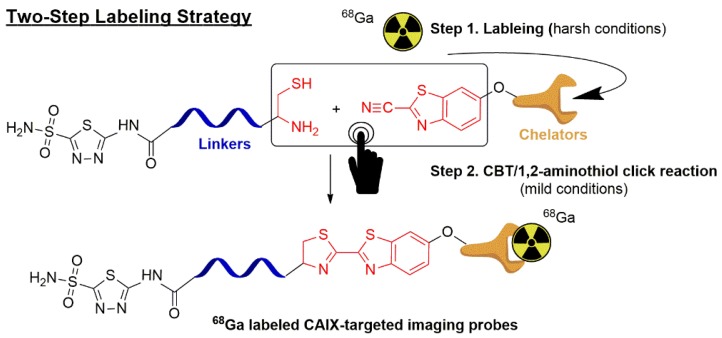
Design of a small library of carbonic anhydrase IX (CAIX) radioligands via a two-step orthogonal labeling concept.

Over the last decade, several CAIX-targeted radioligands based on aromatic sulfonamide pharmacophores, such as acetazolamides (AAZs) and benzene-sulfonamides, have been developed [15,16,17]. By taking advantage of the high binding affinity of aromatic sulfonamide to CAIX, they have been labeled with a variety of radionuclides (i.e., ^18^F, ^64^Cu, ^68^Ga, ^99m^Tc, ^111^In) and evaluated in preclinical CAIX-positive tumor models. However, most of these probes suffered from low tumor uptake, weak selectivity and stability when evaluated in vivo. The development of this type of probe is still in its early stage and current probes have not been optimized yet [18,19]. Comparison of the target selectivity, physicochemical and pharmacodynamic properties of structurally diverse radiolabeled sulfonamides might provide useful information for probe optimization. Thus, we sought to develop an efficient synthetic method to generate a small library of CAIX-targeted compounds by applying a mild and universal two-step orthogonal labeling protocol. ^68^Ga-labeling is usually performed in an aqueous buffer at low pH and high temperature [20,21]. However these harsh labeling conditions are sometimes not suitable for the direct labeling of fragile biomolecules. Our alternative radiochemical strategy is the two-step labeling approach where a bifunctional chelator (BFC) is radiolabeled and then conjugated to a biovector under biologically friendly conditions. Although sulfonamides are not particularly sensitive to the labeling conditions, we decided to opt for a two-step labeling approach in order to facilitate the implementation of a library-based synthesis of our new CAIX PET tracers (Figure 1). The biovectors and the BFCs were prepared separately, to generate diverse compounds without the need to develop and optimize individually their syntheses. BFCs were functionalized with a 2-cyanobenzothiazole (CBT) clickable group, whereas the CAIX ligands were modified with the complementary 1,2-aminothiol functionality.

Several regioselective click reactions, such as the Huisgen’s cycloaddition, the Staudinger ligation, or the inverse electron demand Diels–Alder reaction (IEDDA) can be applied to the two-step radiosynthesis. In this study, we chose to apply the naturally occurring click reaction between 2-cyanobenzothiazole and 1,2-aminothiol because of its orthogonality, biocompatibility, fast kinetics, and metabolic stability of the reagents and product [22,23,24]. To the best of our knowledge, application of this chemistry to ^68^Ga-labeling has not been reported due to the lack of amenable chemical reagents. Therefore, we describe herein the synthesis of three new CBT-functionalized macrocyclic chelators (**1**–**3**) that can be labeled with ^68^Ga. Then we study their conjugation to acetazolamide derivatives (**8** and **9**) via the CBT/1,2-aminothiol click reaction. A small library of six novel ^68^Ga-labeled CAIX-targeted imaging probes was obtained and their stability in phosphate buffered saline (PBS) and in a transchelation challenge assay were assessed.

## 2. Results and Discussions

**Scheme 1:**
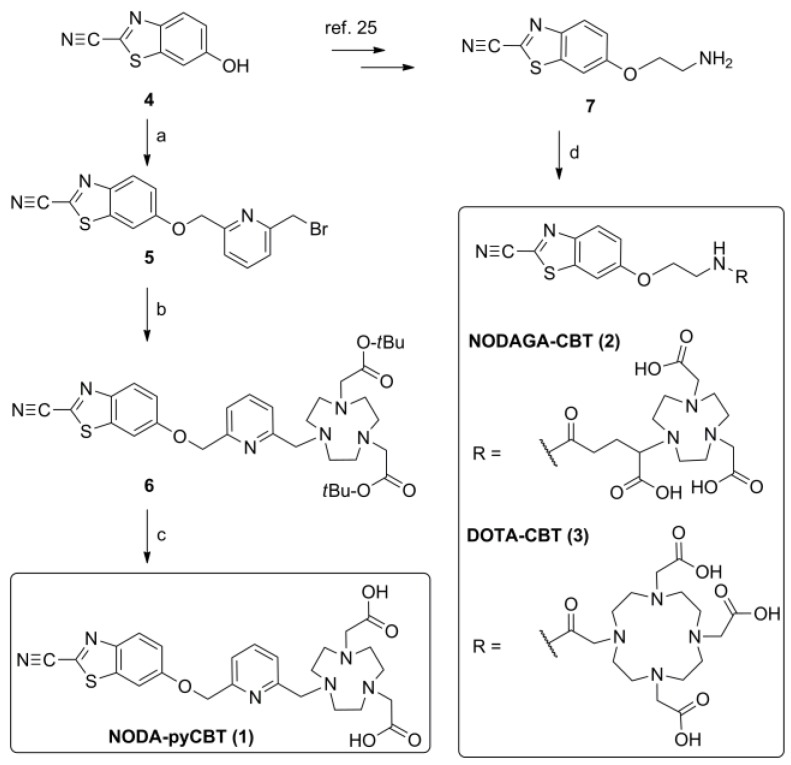
Synthesis of the 2-cyanobenzothiazole (CBT)-bearing chelators (**1**–**3**). Reagents and conditions: (a) 2,6-bis(bromomethyl)pyridine, Cs_2_CO_3,_ THF, 50 °C, 16 h, 69%; (b) di-*tert*-butyl 2,2’-(1,4,7-triazonane-1,4-diyl)diacetate, K_2_CO_3_, KI, ACN, reflux, 16 h, 88%; (c) TFA, thioanisole, DCM, rt, 18 h, 85%, (d) DOTA-NHS or NODAGA-NHS, triethylamine, DMF, rt, 16 h, 29% (for **2**) or 83% (for **3**).

The preparation of the CBT-bearing chelators **1**–**3** is illustrated in Scheme 1. For the synthesis of NODA-pyCBT (**1**), commercially available 2-cyano-6-hydroxy-benzothiazole (**4**) was first *O*-alkylated with 2,6-bis(bromomethyl)pyridine under basic conditions in the presence of cesium carbonate to give the CBT-pyridinyl **5** in 69% yield. *N*-alkylation of the bis-*tert-*butyl NODA chelator with **5** was performed under reflux to give the protected NODA-pyCBT (**6**) in 88% yield. Removal of the *tert-*butyl protecting groups under acidic conditions gave **1** in 85% yield. Notably, thioanisole was used as a cation scavenger to prevent the degradation of the cyano group during the deprotection. For the preparation of **2** and **3**, the amino-CBT intermediate **7** was prepared from **4**, as previously described [25]. Subsequently, **7** was conjugated to DOTA-NHS or NODAGA-NHS under mild basic conditions to give NODAGA-CBT (**2**) or DOTA-CBT (**3**) in 29 and 83% yield, respectively.

**Figure 2:**
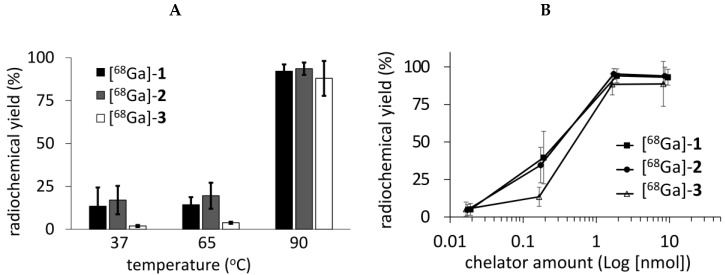
(**A**) Comparison of RCYs for precursors **1**–**3** at 37, 65 and 90 °C. (**B**) Comparison of RCYs when varying amounts of precursor is used. All the reactions were performed at 90 °C by incubation in sodium acetate buffer (0.2 M, pH 5.5) for 15 min.

With the three CBT-functionalized chelators (**1**–**3**) in hand, we next turned our attention to the optimization of the ^68^Ga-labeling conditions. We first evaluated the influence of pH on ^68^Ga-labeling efficiency. The results showed that a nearly quantitative yield of [^68^Ga]-**1** was obtained at the optimal pH of 4.5 to 5.5 (Figure S1). Similarly, the other two CBT-precursors (**2** and **3**) gave excellent ^68^Ga-complexation (>90%) under identical pH conditions. Next, the effect of reaction temperature on radiochemical yields (RCYs) was evaluated. We performed the reaction by incubating the precursors **1**–**3** (~10 nmol) with ^68^GaCl_3_ in sodium acetate buffer (0.2 M, pH 5.5) for 15 min at different temperatures. The RCYs were monitored by radio-high performance liquid chromatography (HPLC). As illustrated in Figure 2A, precursors **1**–**3** were efficiently labeled at 90 °C (88–97% RCYs). However, lowering the temperature was dramatically detrimental to the ^68^Ga-coordination, as RCYs below 25% were observed at 37 and 65 °C for all BFCs. These findings suggest that high temperature is required to provide high yields of ^68^Ga-labeled CBT-derivatives **1**–**3**. To investigate the effect of precursor amount on RCYs, reactions were performed under the optimal pH and temperature conditions defined above. All three precursors could be efficiently labeled with ^68^Ga at a chelator amount equal or superior to 1.5 nmol (Figure 2B). We noticed that **1** and **2** are more prone to complex with ^68^Ga^3+^ at lower concentrations (~0.2 nmol) than the DOTA chelator **3**, which means a higher molar activity could be obtained by using NODA-pyCBT or NODAGA-CBT than precursor **3**. Interestingly, from a structural point of view, similar ^68^Ga-labeling efficiencies of **1** and **2** suggest that the replacement of a carboxylate with a pyridine ring does not alter the chelation of NOTA-type chelators to ^68^Ga^3+^. Furthermore, due to the intrinsic ultraviolet (UV) absorption of the pyridine ring, **1** is more easily monitored than **2** during the compound preparation. Thus, the NODA-pyridine analog could be a viable alternative to NOTA for ^68^Ga labeling.

Assessment of the stability of the complexes ([^68^Ga]-**1**–**3**) in both PBS buffer and through a transchelation challenge study was performed. ^68^Ga-labeled CBTs were incubated in PBS (0.2 M, pH 7.4) in the absence or presence of EDTA (34 mM) at 37 °C for 2 h, and subsequently analyzed by radio-HPLC. All the labeled compounds were stable in the neutral PBS buffer over a period of 2 h (Figure S2). More than 98% of [^68^Ga]-**1**–**3** remained intact, even when challenged by a large excess of EDTA. The high stability of the radiocomplexes warrants their utility in further conjugations with 1,2-aminothiol functionalized compounds.

**Scheme 2:**
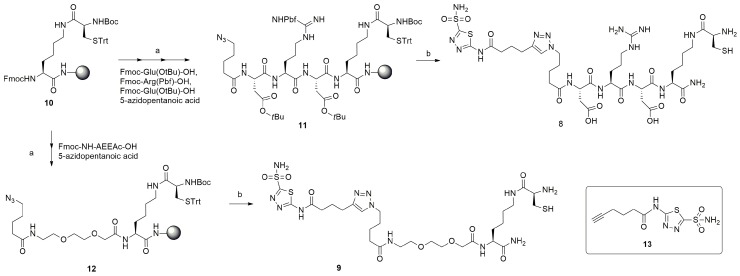
Preparation of **8** and **9** by solid-phase synthesis. Reagents and conditions: (a) Fmoc-SPPS; (b) **13**, CuI, TBTA, sodium ascorbate, DMF, rt, 16 h; (c) TFA/TIPS/H_2_O, rt, 3 h, 32% (for **8**) or 37% (for **9**).

Preparation of two acetazolamide derivatives (**8** and **9**) containing a linker (Asp-Arg-Asp or PEG_2_) and a 1,2-aminothiol moiety were carried out by solid phase synthesis. In general, synthesis of **8** and **9** was initiated by immobilizing a SPPS compatible Fmoc-dipeptide onto a Rink amide MBHA resin (Scheme 2) [25]. Subsequent conjugations with Fmoc-Glu(O*t*Bu)-OH, Fmoc-Arg(Pbf)-OH, Fmoc-Glu(O*t*Bu)-OH and 5-azidopentanoic acid were required for **8**, whereas **9** was prepared according to a similar protocol by using Fmoc-NH-AEEAc-OH instead of the three Fmoc-protected amino acids. The acetazolamide intermediate **13** was then incorporated to the azido-resins to complete the chemical sequence through a Cu(I)-catalyzed Huisgen’s cycloaddition. Cleavage and global deprotection were performed by the treatment of the acetazolamide-resins with a solution of TFA/TIPS/H_2_O (v/v/v = 95/2.5/2.5) to give **8** and **9** in an overall yield of 32% and 37%, respectively, after HPLC purification based on initial resin loading. The pure acetazolamides **8** and **9** were then applied to the preparation of the ^68^Ga-CAIX probes ([^68^Ga]-**L14-L16**).

**Scheme 3:**
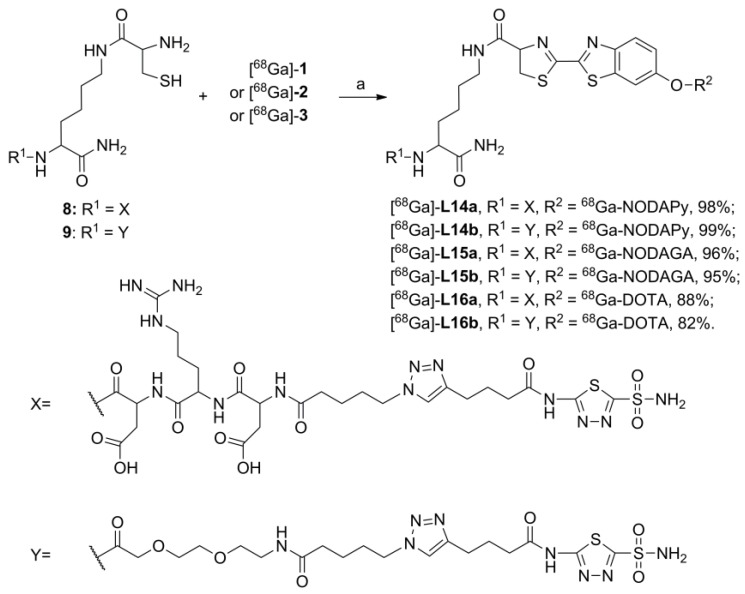
Synthesis of [^68^Ga]-**14** to [^68^Ga]-**16** via CBT/1,2-aminothiol click reaction. Reagents and conditions: (a) TCEP·HCl, PBS, pH 9.0, 37 °C, 20 min.

**Figure 3:**
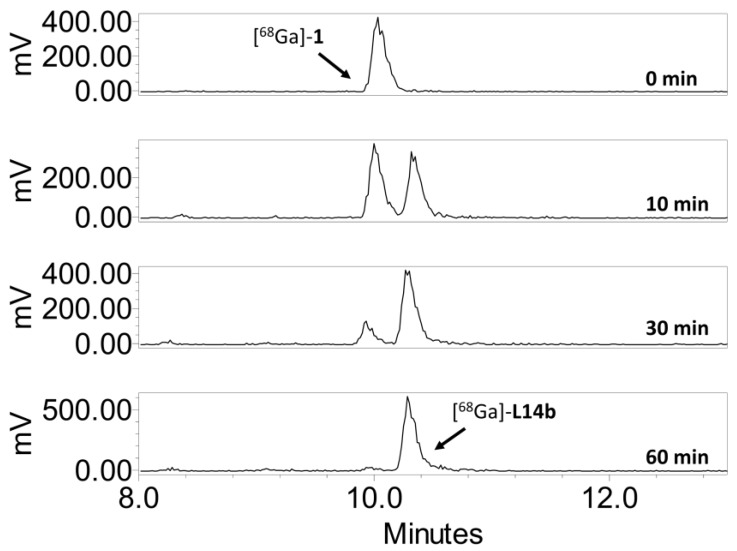
Radio-high performance liquid chromatography (HPLC) monitoring of the progress of click reaction between [^68^Ga]-**1** and **9**. The retention time of [^68^Ga]-**1** and [^68^Ga]-**L14b** are 10.0 and 10.3 min, respectively (HPLC system A).

The conjugates [^68^Ga]-**L14-L16** were obtained by CBT/1,2-aminothiol click ligation of the ^68^Ga-labeled BFCs ([^68^Ga]-**1**–**3)** and the two acetozalamides (**8** and **9**) (Scheme 3). The efficiency of click reaction between [^68^Ga]-**1** and **9** to form [^68^Ga]-**L14b** was first tested at pH 7.4. At different time points, the reaction was quenched by addition of 10% acetic acid and analyzed by radio-HPLC to monitor the progress of the reaction. As illustrated in Figure 3, the formation of [^68^Ga]-**L14b** could already be observed at 10 min (RCY of 44%), demonstrating the fast kinetics of the click reaction. Identity of the clicked product ([^68^Ga]-**L14b**) was confirmed by HPLC comparison of a non-radioactive standard (Figure S3). Longer reaction time resulted in improved radiochemical yields. Surprisingly, the conjugation of **8** to [^68^Ga]-**1** was not as efficient as the click reaction between **9** and [^68^Ga]-**1** (Figure S4). RCYs of 54 and 78% were obtained when [^68^Ga]-**1** was treated with **8** for 30 and 60 min (Table 1, entry 1 and 2), as compared to 81 and 99% for the reaction between [^68^Ga]-**1** and **9**. Similar observations have previously been reported for other 1,2-aminothiol substrates, where the click reaction rate was presumably affected by the differences of chemical structures, configurations and electronic distribution [26,27]. Although a high RCY (98%) could be reached by extending the reaction time to 180 min (Table 1, entry 3), it was not practical for ^68^Ga-labeling considering its short half-life. We previously demonstrated that the ligation between 2-cyano-6-hydroxybenzothiazole and l-cysteine in PBS at pH 9.0 is 4-times more efficient than at pH 7.4 [27]. Thus, we evaluated the click reaction between [^68^Ga]-**1** and **8** at pH 9.0 in PBS. To our delight, the RCY of [^68^Ga]-**L14a** was significantly improved to 85% after 30 min reaction time (as opposed to 54% at pH 7.4), and a nearly quantitative yield was observed after 60 min (Table 1, entries 4 and 5). We also checked if a higher concentration of 1,2-aminothiol-AAZ would accelerate the reaction. An increase in the amount of **8** from 10 to 25 nmol resulted in an excellent RCY (99%) after 20 min incubation time (Table 1, entry 6). Then, we applied the above slightly basic conditions to the ligation of the two other ^68^Ga-CBTs ([^68^Ga]-**2** and [^68^Ga]-**3**) with AAZs (**8** and **9**). [^68^Ga]-**L14b**, [^68^Ga]-**L15a** and [^68^Ga]-**L15b** were successfully obtained with high RCYs (95–99%) within 20 min. These radiochemical products were amenable for direct utilization in preclinical tests without further purification. Lower RCYs of 88 and 82% were found for [^68^Ga]-**L16a** and [^68^Ga]-**L16b**, respectively. It might be explained by a weaker complexation of ^68^Ga^3+^ in the DOTA chelator (the stability constants (log *K*_ML_) of Ga-DOTA and Ga-NOTA are 26 and 31, respectively), and therefore a small amount of free ^68^Ga was released from the [^68^Ga]-**3** complex during the conjugation reaction [28,29]. It confirms that BFCs **1** and **2** are preferred over BFCs **3** for ^68^Ga-labeling, since the ^68^Ga complexation in **1** and **2** is more efficient and stable than in **3** (Figure 2B and Figure S5). Nevertheless, our DOTA-based BFC **3** could be very valuable for labeling with other radiometals, such as ^90^Y and ^177^Lu.

Stability studies were performed by incubation of our ^68^Ga-labeled CAIX-targeted probes ([^68^Ga]-**L14-L16)** in PBS at 37 °C for 2 h. All our compounds showed excellent stability, with more than 99% of intact radioligand after 2 h incubation. It indicates that our radiolabeled compounds [^68^Ga]-**L14-L16** were not subject to radiolytic degradation over this time period (Table 2). Lipophilicity of the ^68^Ga-labeled ligands was determined by measurement of the LogD_7.4_. All our radiolabeled compounds were hydrophilic and water-soluble, and therefore they are more prone to be cleared via the kidney [30]. In general, replacing the PEG_2_ linker by a peptide inker (Asp-Arg-Asp) had little effect on the molecular lipophilicity, suggesting that this charged peptide linker can be used for a comparison of the overall charge effects with its corresponding PEG surrogate on in vivo pharmacokinetics. In contrary, the Log D_7.4_ values were found to be affected when using different ^68^Ga-labeling chelators. For instance, [^68^Ga]-**L14a** and [^68^Ga]-**L14b** exhibited significantly higher hydrophobicity than the other two series compounds. Those compounds with various physicochemical properties could be used for a comparison of their in vivo effects to guide the direction of probe optimization.

## 3. Materials and Methods

### 3.1. General Information

All chemicals were obtained from commercial suppliers and used without further purification. NODAGA-NHS ester and DOTA-NHS ester were obtained from CheMatech (Dijon, France). All solvents were anhydrous grade unless indicated otherwise. ^68^Ga was obtained from a ^68^Ga/^68^Ge generator (IGG-100; Eckert and Ziegler Europe, Berlin, Germany). Reactions were magnetically stirred and monitored by thin-layer chromatography on Merck aluminum-backed pre-coated plates (Silica gel 60 F254) (Quebec, QC, Canada), and visualized with ultraviolet light or by staining with 10% phosphomolybdic acid in neat ethanol. Flash chromatography was performed on silica gel of 40–63 µm particle size. Concentration refers to rotary evaporation. Reverse-phase high-performance liquid chromatography (HPLC) was carried out on a Waters^®^ 2659 series system (Etten-Leur, The Netherlands) equipped with a diode array detector and a radio-detector. Nuclear magnetic resonance (NMR) spectra were recorded in DMSO-*d*6, D_2_O, CDCl_3_ or CD_3_OD on diluted solutions on a Bruker AVANCE 400 (Leiderdorp, Leiden, The Netherlands) at ambient temperature. Chemical shifts are given as *δ* values in ppm and coupling constants *J* are given in Hz. The splitting patterns are reported as s (singlet), d (doublet), t (triplet), m (multiplet), dd (doublet of doublets) and br (broad signal). Low-resolution electrospray ionization (ESI) mass spectra were recorded on a TSQ Quantum Ultra™ triple quadrupole mass spectrometer from Thermo Fisher Scientific^®^ (Bleiswijk, Lansingerland, The Netherlands). Fmoc-based solid-phase peptide synthesis (SPPS) was conducted on an C.S. Bio CS136 automated peptide synthesizer (Menlo Park, CA, USA).

### 3.2. High-Performance Liquid Chromatography (HPLC) Conditions for Analysis

The analyses of reaction were performed by HPLC on an analytical RP-C18 column (5 μm, 4.6 × 250 mm, Phenomenex Aqua^®^, Torrance, CA, USA) at a flow rate of 1 mL/min. The UV signal was recorded at wavelength of 254 nm. The following solvents and eluting gradients were used: solvent A = 0.1% trifluoroacetic acid (TFA) in water (v/v); solvent B = 0.1% TFA in acetonitrile (v/v). HPLC system A, a gradient of solvent A and B: t = 0–20 min, 95 to 5% A; t =20–23 min, 5% A; HPLC system B, a gradient of solvent A and B: t = 0–25 min, 95 to 55% A; t = 25–27 min, 55 to 0% A; t = 27–30 min, 0% A were applied.

### 3.3. HPLC Conditions for Purification

The HPLC purifications were performed on a Phenomenex semi preparative RP-C18 column (Aqua^®^, 5 μm, 10 × 250 mm) at a flow rate of 3.0 mL/min. A gradient of solvent A and B (t = 0–5 min, 90% A; t = 5–30 min, 90 to 0% A) was applied.

### 3.4. Chemistry and Radiolabeling

*6-((6-(Bromomethyl)pyridin-2-yl)methoxy)benzo[d]thiazole-2-carbonitrile* (**5**). To a solution of 2,6-bis(bromomethyl)pyridine (460 mg, 1.74 mmol) and 2-cyano-6-hydroxybenzothiazole (200 mg, 1.14 mmol) in THF (50 mL) was added Cs_2_CO_3_ (450 mg, 1.38 mmol). The reaction was heated at 50 °C for 16 h then cooled to rt and filtered. The filtrate was concentrated and purified by flash chromatography (hexanes/EtOAc, 6:4) to afford **5** as a white solid (284 mg, 69%). ^1^H NMR (400 MHz, CDCl_3_): *δ* 8.10 (d, 1H, *J =* 9.2 Hz), 7.76 (t, 1H, *J =* 7.6 Hz), 7.40–7.47. (m, 3H), 7.34 (dd, 1H, *J =* 2.4, 9.2 Hz), 5.30 (s, 2H), 4.58 (s, 2H). ESI-MS: *m/z* 360.0 [M + H]^+^.

*Di-tert-butyl 2,2’-(7-((6-(((2-cyanobenzo[d]thiazol-6-yl)oxy)methyl)pyridin-2-yl)met-hyl)-1,4,7-triazonane-1,4-diyl)diacetate* (**6**). To a mixture of di-*tert*-butyl 2,2’-(1,4,7-triazonane-1,4-diyl)diacetate (49 mg, 0.14 mmol), K_2_CO_3_ (76 mg, 0.52 mmol) and KI (0.3 mg, 1.81 μmol) in acetonitrile (2 mL) was added dropwise a solution of **5** (50 mg, 0.14 mmol) in acetonitrile (3 mL). The reaction was allowed to stir at rt for 1 h, then refluxed for 16 h. The reaction was cooled to rt, filtered and concentrated to give **6** as a yellow oil (77 mg, 88%). The product was directly used without further purification. ^1^H NMR (400 MHz, CDCl_3_): δ 8.07 (d, 1H, *J* = 9.2 Hz), 7.68 (t, 1H, *J* = 7.6 Hz), 7.51 (br, 1H), 7.42 (br, 1H), 7.33 (m, 2H), 5.26 (m, 2H), 3.86 (s, 2H), 3.30 (s, 4H), 2.87 (m, 12H), 1.43 (s, 18H). ESI-MS: *m/z* 637.3 [M + H]^+^, 659.3 [M + Na]^+^.

*2,2’-(7-((6-(((2-Cyanobenzo[d]thiazol-6-yl)oxy)methyl)pyridin-2-yl)methyl)-1,4,7-triazonane-1,4-diyl)diacetic Acid* (NODA-pyCBT, **1**). TFA (1 mL) was added dropwise to a solution of crude **6** (24 mg, 37.67 μmol), thioanisole (20%) in 1 mL DCM at 0 °C and allowed to stir at rt for 18 h. The reaction mixture was concentrated under reduced pressure and purified by semi preparative HPLC to yield **1** as a yellow solid (17 mg, 85%). ^1^H NMR (400 MHz, CD_3_OD): *δ* 8.12 (d, 1H, *J =* 9.2 Hz), 7.95 (t, 1H, *J =* 7.6 Hz), 7.80 (m, 1H), 7.68 (m, 1H), 7.60 (m, 1H), 7.44 (m, 1H), 5.40 (s, 2H), 4.47 (s, 2H), 3.55 (m, 4H), 3.24 (s, 6H), 2.94 (s, 6H). High-resolution mass spectrometry (HRMS) (ESI): *m/z* calcd. for [C_25_H_28_N_6_O_5_S + H]^+^ 525.1920, found: 525.1917. The compound purity was determined by UV-HPLC (254 nm), showing greater than 95% (Figure S7).

*2,2’-(7-(1-Carboxy-4-((2-((2-cyanobenzo[d]thiazol-6-yl)oxy)ethyl)amino)-4-oxobutyl)-1,4,7-triazonane-1,4-diyl)diacetic Acid* (NODAGA-CBT, **2**). To a solution of **7** (8 mg, 0.02 mmol) in DMF (1 mL) was added 33 μL of triethylamine (0.24 mmol). Subsequently, a solution of NODAGA-NHS ester (15 mg, 0.02 mmol) in 0.15 mL of DMF was added to the reaction mixture. The vial was placed under a positive pressure of argon and the solution was stirred for 16 h at rt. Solvent was removed under vacuum and the residue was dissolved in H_2_O/ACN (1:1) and purified by semi-preparative HPLC to yield **2** as a pale-yellow solid upon lyophilization (4 mg, 29%). ^1^H NMR (400 MHz, CD_3_OD): *δ* 8.07 (d, 1H, *J =* 9.2 Hz), 7.68 (m, 1H), 7.33 (dd, 1H, *J* = 2.4, 9.2 Hz), 4.33–4.36 (m, 2H), 3.68–3.88 (m, 6H), 3.43 (m, 1 H), 2.89–3.25 (m, 12H), 2.36–2.48 (m, 2H), 1.91–2.08 (m, 2H). ESI-MS: *m/z* 595.3 [M + H_3_O]^+^. The compound purity was determined by UV-HPLC (254 nm), showing greater than 95% (Figure S7).

*2,2’,2’’-(10-(2-((2-((2-Cyanobenzo[d]thiazol-6-yl)oxy)ethyl)amino)-2-oxoethyl)-1,4,7,10-tetraazacyclododecane-1,4,7-triyl)triacetic Acid* (DOTA-CBT, **3**). **3** was prepared according to the method described above for **2**. Semi-preparative HPLC purification provided 3 as a white solid upon lyophilization (12 mg, 83%). ^1^H NMR (400 MHz, CD_3_OD): *δ* 7.97–8.02 (m, 1H), 7.59–7.61 (m, 1H), 7.27–7.33 (m, 1H), 4.33 (t, 2H, *J* = 4.8 Hz), 3.69–4.1 (m, 10 H), 3.58 (t, 2H, *J* = 4.8 Hz), 3.2–3.44 (m, 14H). ESI-MS: *m/z* 624.4 [M + H_3_O]^+^. The compound purity was determined by UV-HPLC (254 nm), showing greater than 95% (Figure S7).

*N-(5-sulfamoyl-1,3,4-thiadiazol-2-yl)hex-5-ynamide* (**13**). The 5-hexynoic acid (100 mg, 0.89 mmol, 3.1 equiv.) was treated with an excess of thionyl chloride (2 mL, 27.56 mmol) at 0 °C, and the reaction mixture was heated to 70 °C and stirred for 1 h. 5-Hexynoyl chloride was obtained by evaporation of the excess of thionyl chloride and dried under vacuum. A solution of 5-amino-1,3,4-thiadiazole-2-sulfonamide (50 mg, 0.28 mmol, 1.0 equiv.) in anhydrous DMF (2 mL) was slowly added into 5-hexynoyl chloride at 0 °C under N_2_. The reaction mixture was warmed to rt and stirred for 48 h. The reaction mixture was then concentrated and purified by flash chromatography (with a gradient of EtOAc/Hexanes = 1:4 to 1:1 to 100% EtOAc; silica gel) to give the product as an off-white solid (42 mg, 55%). ^1^H NMR (400 MHz, DMSO-d6): *δ* 13.02 (s, 1H), 8.31 (s, 2H), 2.82 (t, 1H, *J* = 2.4 Hz), 2.63 (t, 2H, *J* = 7.2 Hz), 2.21–2.25 (m, 2H), 1.78–1.81 (m, 2H). ESI-MS: *m/z* 297.1 [M + Na]^+^.

*K(C)-DRD-AAZ* (**8**). Compound **8** was prepared by solid-phase synthesis in a 10 mL reaction vessel (Chemglass^®^, Vineland, NJ, USA). All the reactions were performed at rt with agitation at 150 rpm. The washing steps were performed with DMF (5 mL × 2) and DCM (5 mL × 2). The capping was carried out by using Ac2O (94.5 μL, 1.00 mmol) in DMF (5 mL). Before the first amino acid coupling, Rink amide MBHA resin (100 mg, 0.65 mmol/g) was preswollen with DMF (5 mL) for 1 h, and then treated with 20% piperidine in DMF (5 mL) for 1 h to remove the Fmoc-protecting group on resin. A mixture of Fmoc-Lys[N-Boc-Cys(Trt)]-OH (159 mg, 0.19 mmol), HBTU (76 mg, 0.32 mmol), Oxymapure (46 mg, 0.32 mmol), and DIPEA (83 μL, 0.65 mmol) in DMF (5 mL) was added to the resin and agitated for 2 h. After a washing/capping sequence, the Fmoc-l-Lys(N-Boc-l-Cys(Trt))-resin was obtained. Subsequent couplings with Fmoc-l-Asp(tBu)-OH (80 mg, 0.19 mmol), Fmoc-l-Arg(Pbf)-OH (126 mg, 0.19 mmol), Fmoc-l-Asp(*t*Bu)-OH (80 mg, 0.19 mmol) and 5-azidopentanoic acid (28 mg, 0.19 mmol) were accomplished according to the same experimental protocol to give the azido-peptidic resin. The coupling reactions were performed in DMF with HBTU (5.0 equiv.), OxymaPure (5.0 equiv.) and DIPEA (10.0 equiv.) for 2 h. Fmoc deprotection was achieved by treatment of the resin with piperidine (20%) in DMF for 0.5 h. The amide formation and Fmoc deprotection were monitored by Kaiser test. Double couplings or deprotection were performed when the reaction was not completed. The click reaction was carried out by mixing the azido-peptidic resin, 13 (53 mg, 0.19 mmol), CuI (4 mg, 0.02 mmol), TBTA (10 mg, 0.02 mmol) and sodium ascorbate (34 mg, 0.20 mmol) in DMF (5 mL) for 16 h. Cleavage from the resin and deprotection of the AAZ analog were conducted by treatment of the resin with TFA/TIPS/H_2_O (3 mL, v/v/v = 95/2.5/2.5) at rt for 3 h. The cleavage cocktail was concentrated, washed with ice-cold ether (12 mL × 3) and the residue purified by semi-preparative HPLC to give **8** as a white solid (22 mg, 32%). ^1^H NMR (400 MHz, D_2_O): *δ* 7.84 (s, 1H), 4.67–4.73 (m, 2H), 4.29–4.36 (m, 3H), 4.23–4.27 (m, 1 H), 4.17 (t, 1H, *J =* 6.0 Hz), 3.17–3.35 (m, 4H), 3.06 (dd, 1H, *J =* 4.0, 5.6 Hz), 2.88–3.03 (m, 3H), 2.79–2.88 (m, 4H), 2.66–2.70 (m, 2H), 2.31 (t, 2H, *J =* 7.6 Hz), 2.12–2.18 (m, 2H), 1.69–1.91 (m, 6H), 1.51–1.66 (m, 6H), 1.33–1.45 (m, 2H). ESI-MS: *m/z* 1034.37 [M + H]^+^. The compound purity was determined by UV-HPLC (254 nm), showing greater than 95% (Figure S7).

*K(C)-PEG-AAZ* (**9**). The preparation of **9** was similar to the preparation of **8**. The synthesis was started by loading Fmoc-Lys[*N*-Boc-Cys(Trt)]-OH onto Rink amide MBHA resin (100 mg, 0.65 mmol/g), followed by subsequent conjugation with Fmoc-AEEAc-OH (75 mg, 0.19 mmol), 5-azidopentanoic acid (28 mg, 0.19 mmol) and 13 (53 mg, 0.19 mmol). After cleavage and deprotection, the crude product was washed with cold-ether and purified by HPLC to give **9** as white solid (19 mg, 37%). ^1^H NMR (400 MHz, D_2_O): *δ* 7.68 (s, 1H), 4.12–4.18 (m, 4 H), 3.96 (s, 2H), 3.53 (dd, 4H, *J =* 4.4, 5.2 Hz), 3.46 (t, 2H, *J =* 5.2 Hz), 3.23 (dd, 2H, *J =* 4.8, 5.6 Hz), 3.01–3.18 (m, 4 H), 2.67 (d, 2H, *J =* 7.2 Hz), 2.51 (d, 2H, *J =* 7.2 Hz), 2.10 (d, 2H, *J =* 7.2 Hz), 1.94–1.99 (m, 2H), 1.63–1.68 (m, 3H), 1.55–1.58 (m, 1H), 1.38–1.40 (m, 4H), 1.19–1.27 (m, 2H). ESI-MS: *m/z* 815.27 [M + Na]^+^. The compound purity was determined by UV-HPLC (254 nm), showing greater than 95% (Figure S7).

Radiolabeling of NODA-pyCBT (**1**). ^68^GaCl_3_ was eluated from the ^68^Ge/^68^Ga-Generator as an aqueous solution containing 0.05 M HCl and 3.0 M NaCl. ^68^GaCl_3_ (200 μL, 115 MBq) was added to a solution of 1 (9.5 nmol) in DMSO (1 μL) and sodium acetate buffer (0.2 M, pH 5.5, 700 μL). The mixture was incubated at 90 °C with shaking at 700 rpm for 15 min. The reaction conversion was monitored by radio-TLC and a solution of NH_4_OAc/MeOH (1.0 M, v/v = 1:1) was used as mobile phase. The reaction mixture was cooled for 5 min, and then passed through a C-18 light cartridge. The cartridge was washed with water (10 mL) to remove unchelated-^68^Ga and followed by washed with ethanol (1 mL) to give [^68^Ga]-**1**. [^68^Ga]-**1** was obtained with a radiochemical yield of 85% (decay corrected) and a molar activity of 8.9 MBq/nmol after purification. The radiochemical purity of [^68^Ga]-**1** was determined by radio-HPLC, showing greater than 99%. The retention time of [^68^Ga]-**1** was 10.0 min (HPLC system A).

Radiolabeling of NODAGA-CBT (**2**). [^68^Ga]-**2** (8.6 nmol) was labeled with ^68^GaCl_3_ (84 MBq) under similar conditions as previously described for [^68^Ga]-**1**. [^68^Ga]-**2** was obtained with a radiochemical yield of 94% (d. c.) and a molar activity of 6.9 MBq/nmol after purification. The retention time of [^68^Ga]-**2** was 11.1 min (HPLC system A) and its radiochemical purity was greater than 95%.

Radiolabeling of DOTA-CBT (**3**). [^68^Ga]-**3** (8.2 nmol) was labeled by ^68^GaCl_3_ (98 MBq) by using the similar conditions of preparing [^68^Ga]-**3**. The [^68^Ga]-**3** was obtained in the radiochemical yield of 85% (d. c.) with molar activity of 7.1 MBq/nmol after purification. The radiochemical purity of [^68^Ga]-**3** was determined by radio-HPLC, showing greater than 99%. The retention time of [^68^Ga]-**3** was 10.2 min (HPLC system A).

Conjugation of ^68^Ga-labeled bifunctional chelators (BFCs) ([^68^Ga]-**1**–**3**) and acetazolamides (AAZs) (**8**–**9**). Compound **8** (26 μg, 25 nmol) or **9** (20 μg, 25 nmol), TCEP·HCl (8 μg, 28 nmol) in PBS (0.2 M, pH 9.0, 400 μL) were mixed in a 1.5 mL centrifuge tube at rt. To the mixture, ^68^Ga-labeled precursors (2 nmol, 10–20 MBq) in 100 μL of EtOH, was added, and the reaction was incubated at 37 °C with agitation (700 rpm) for 20 min. The radiochemical purity (RCP) and retention time (*t*_R_) of products [^68^Ga]-**L14a**-**L16b** were analyzed by radio-HPLC (Table 3 and Figure S5).

### 3.5. Determination of LogD_7.4_

Distribution coefficients (LogD_7.4_ values) were determined by a shake-flask method. Sample containing the radioligand in 5 μL PBS (pH 7.4) was added to a vial containing 595 μL PBS (pH 7.4) and 600 μL n-octanol. The vial was vortexed vigorously and then centrifuged for 10 min for phase separation. Samples of the octanol (200 μL) and aqueous (200 μL) phases were taken and counted by γ-counter. LogD_7.4_ value was calculated by using the equation: LogD_7.4_ = log [(counts in octanol phase)/(counts in aqueous phase)]. All the experiments were performed in triplicates.

### 3.6. Stability and Challenge Studies

For the stability and challenge tests, the radiolabeled samples (~2 MBq) were mixed with 300 μL of PBS (0.1 M, pH 7.4) or a PBS/EDTA solution (34 mM EDTA in PBS), respectively. After 2 h incubation at 37 °C, the samples were analyzed by radio-HPLC (HPLC system B for [^68^Ga]-**L16b**; HPLC system A for other compounds).

## 4. Conclusions

We have developed a two-step orthogonal labeling method to prepare a small library of ^68^Ga-labeled CAIX ligands via a CBT/1,2-aminothiol click reaction. Three novel CBT-functionalized chelators (**1**–**3**) were synthesized and efficiently labeled with ^68^Ga while retaining their clickable functionality. The physicochemical properties, such as lipophilicity, solubility and overall charges, of a NOTA-type chelator could be manipulated by modification or replacement of one of the carboxylate arms on NOTA. Replacement of a carboxylate with a pyridine ring has no detrimental effect on the chelation of ^68^Ga^3+^, but it enhances its hydrophobicity. Six new ^68^Ga-labeled CAIX-targeted molecules were successfully prepared under optimal conditions by cross-ligation between our three ^68^Ga-labeled chelators and two acetazolamide derivates. The products exhibited high radiochemical purity and in vitro stability, allowing direct application for further biological evaluations. Our chemistry and materials provide a high degree of versatility to develop new target-specific imaging or therapeutic probes, but can also be considered for pretargeting applications. An in vitro receptor binding affinity assay, a cell internalization assay, an in vivo biodistribution and μPET studies are currently underway in our laboratory to explore the effects of the molecular composition on the physicochemical properties of our CAIX radioligands.

## Figures and Tables

**Table 1:** Evaluation of the click reaction between [^68^Ga]-**1** and **8** under varying conditions.

Entry	8 (nmol)	Buffer pH	Time (min)	RCY (%) ^*a*^
1	10	7.4	30	54
2	10	7.4	60	78
3	10	7.4	180	98
4	10	9.0	30	85
5	10	9.0	60	99
6	25	9.0	20	99

***^a^*** Radiochemical yield (RCY) of [^68^Ga]-**L14a** was determined by radio-HPLC.

**Table 2:** Determination of LogD_7.4_ and stability of [^68^Ga]-**L14-L16.**

Compound	Stability (%) *^a^*	Log D_7.4_
[^68^Ga]-**L14a**	>99	−1.95 ± 0.03
[^68^Ga]-**L14b**	>99	−2.03 ± 0.10
[^68^Ga]-**L15a**	>99	−2.68 ± 0.02
[^68^Ga]-**L15b**	>99	−2.71 ± 0.05
[^68^Ga]-**L16a**	>99	−3.20 ± 0.06
[^68^Ga]-**L16b**	>99	−3.29 ± 0.14

***^a^*** The stability tests were performed in phosphate buffered saline (PBS) at 37 °C for 2 h. Results are expressed as percentage (%) of intact ligand after incubation.

**Table 3:** Conjugation of ^68^Ga-labeled bifunctional chelators (BFCs) and acetazolamides (AAZs) through the click reaction.

^68^Ga-Labeled BFCs	AAZs	Products	RCP (%)	*t*_R_ (min)/ HPLC System
[^68^Ga]-**1**	8	[^68^Ga]-**L14a**	98	9.4 / A
[^68^Ga]-**1**	9	[^68^Ga]-**L14b**	99	10.3 / A
[^68^Ga]-**2**	8	[^68^Ga]-**L15a**	96	9.9 / A
[^68^Ga]-**2**	9	[^68^Ga]-**L15b**	95	10.8 / A
[^68^Ga]-**3**	8	[^68^Ga]-**L16a**	88	9.5 / A
[^68^Ga]-**3**	9	[^68^Ga]-**L16b**	82	18.5 / B *

* The retention time of [^68^Ga]-**3** is 16.7 min in HPLC system B.

## Supplementary Materials

The supplementary materials are available online.
