# Supplementary material for: A Flexible Synthesis of 68Ga-Labeled Carbonic Anhydrase IX (CAIX)-Targeted Molecules via CBT/1,2-Aminothiol Click Reaction
Source: Molecules. 2018 Dec 21;24(1):23. doi: 10.3390/molecules24010023 (PMC6337199; doi:10.3390/molecules24010023)
Supplement: Supplementary file 1 [file molecules-24-00023-s001.pdf]

Supporting Information for

A Flexible Synthesis of ^{68}Ga-Labeled Carbonic Anhydrase IX (CAIX)-Targeted Molecules via CBT/1,2-Aminothiol Click Reaction

Kuo-Ting Chen [a], Kevin Nguyen [b], Christian Ieritano [b], Feng Gao [b] and Yann Seimbille [a,b]

[a] Erasmus MC, University Medical Center Rotterdam, Department of Radiology and Nuclear Medicine, Wytemaweg 80, 3015 CN Rotterdam, The Netherlands. E-mail: y.seimbille@erasmusmc.nl

[b] TRIUMF, Life Sciences Division, 4004 Wesbrook Mall, Vancouver, V6T 2A3, British Columbia, Canada.

**Contents**	**Pages**
Figure S1. Radio-HPLC monitoring of the ^{68}Ga-labeling of **1** at different pH values.	1
Figure S2. Determination of the radiochemical purity and stability of **[^{68}Ga]-1** (A), **[^{68}Ga]-2** (B) and **[^{68}Ga]-3** (C) by radio-HPLC.	2
Figure S3. Identification of **[^{68}Ga]-L14b**.	4
Figure S4. Radio-HPLC monitoring of the progress of click reaction between **[^{68}Ga]-1** and **8**.	5
Figure S5. Radio-HPLC monitoring of the click reaction between ^{68}Ga-CBTs and AAZs.	6
Figure S6. Determination of the stability of **[^{68}Ga]-L14-L16** in PBS by radio-HPLC.	8
Figure S7. Determination of the purity of **1** (A), **2** (B), **3** (C), **8** (D) and **9** (E) by HPLC.	9
Figure S8. ^{1}H-NMR Spectra of compound **1** (400 MHz, CD_3OD)	10
Figure S9. ^{1}H-NMR Spectra of compound **2** (400 MHz, CD_3OD).	11
Figure S10. ^{1}H-NMR Spectra of compound **3** (400 MHz, CD_3OD).	12
Figure S11. ^{1}H-NMR Spectra of compound **5** (400 MHz, $CDCl_3$).	13
Figure S12. ^{1}H-NMR Spectra of compound **6** (400 MHz, $CDCl_3$).	14
Figure S13. ^{1}H-NMR Spectra of compound **8** (400 MHz, D_2O).	15
Figure S14. ^{1}H-NMR Spectra of compound **9** (400 MHz, D_2O).	16
Figure S15. ^{1}H-NMR Spectra of compound **13** (400 MHz, DMSO-*d*6).	17

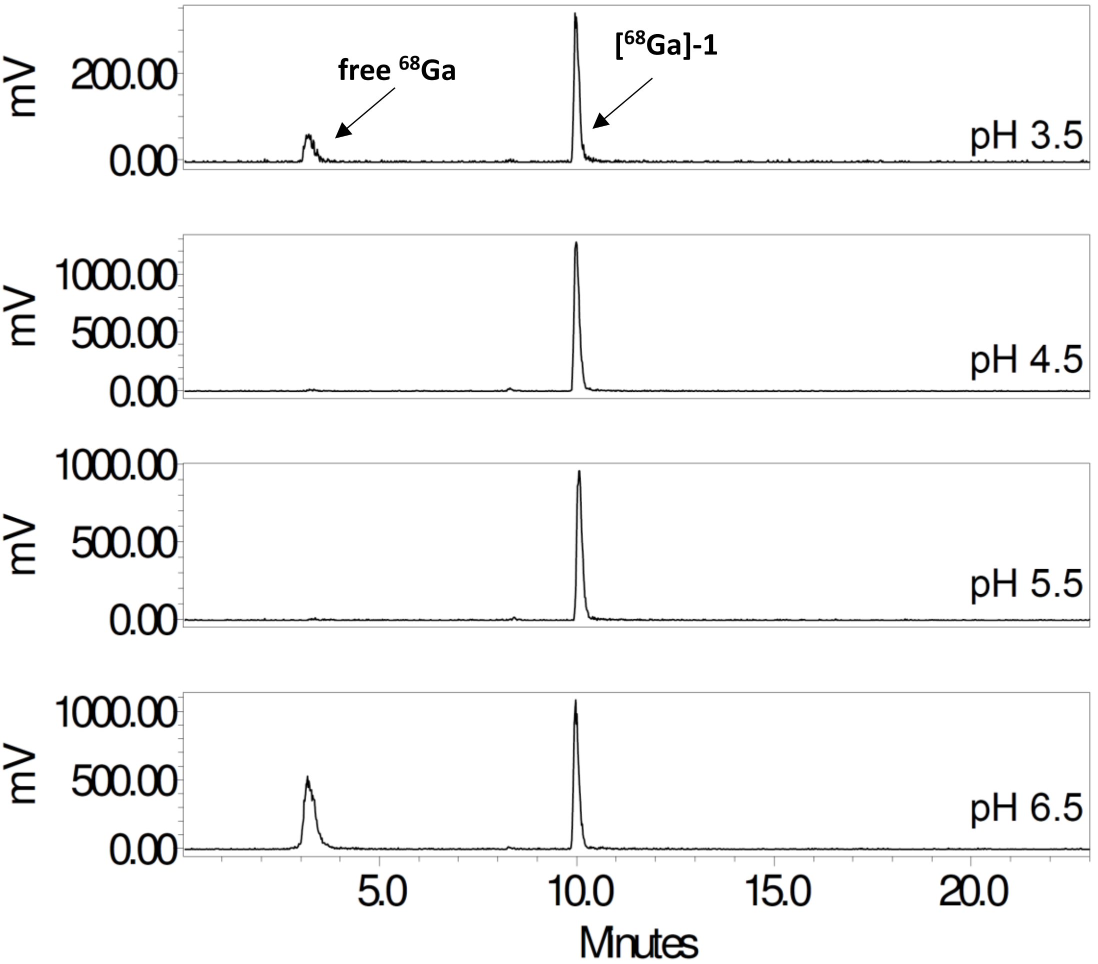

Figure S1. Radio-HPLC monitoring of the ^{68}Ga-labeling of **1** at different pH values. The reaction was performed in sodium acetate buffer at 90 ^{o}C for 15 min with agitation (700 rpm). The RCYs were determined by radio-HPLC (system A). The retention time of free ^{68}Ga and **[^{68}Ga]-1** were 3.3 and 10.0 min, respectively. The RCYs of the reactions at pH 3.5, 4.5, 5.5 and 6.5 were 78, 100, 100 and 51%, respectively.

(A) **[⁶⁸Ga]-1**

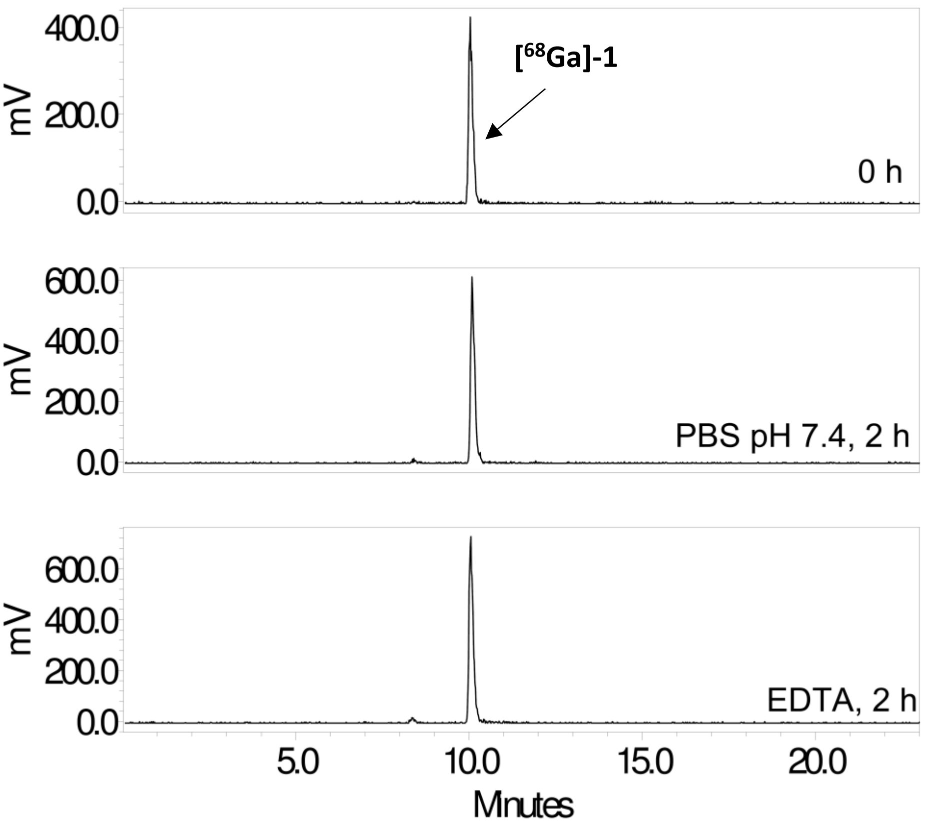

(B) **[⁶⁸Ga]-2**

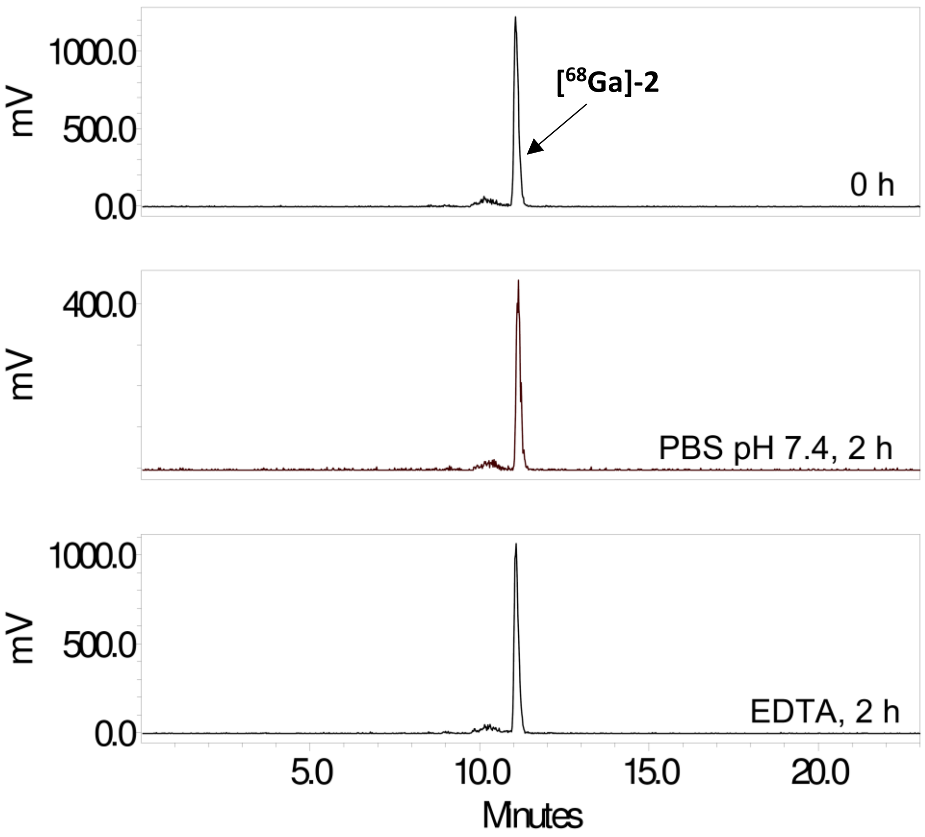

(C) **[^{68}Ga]-3**

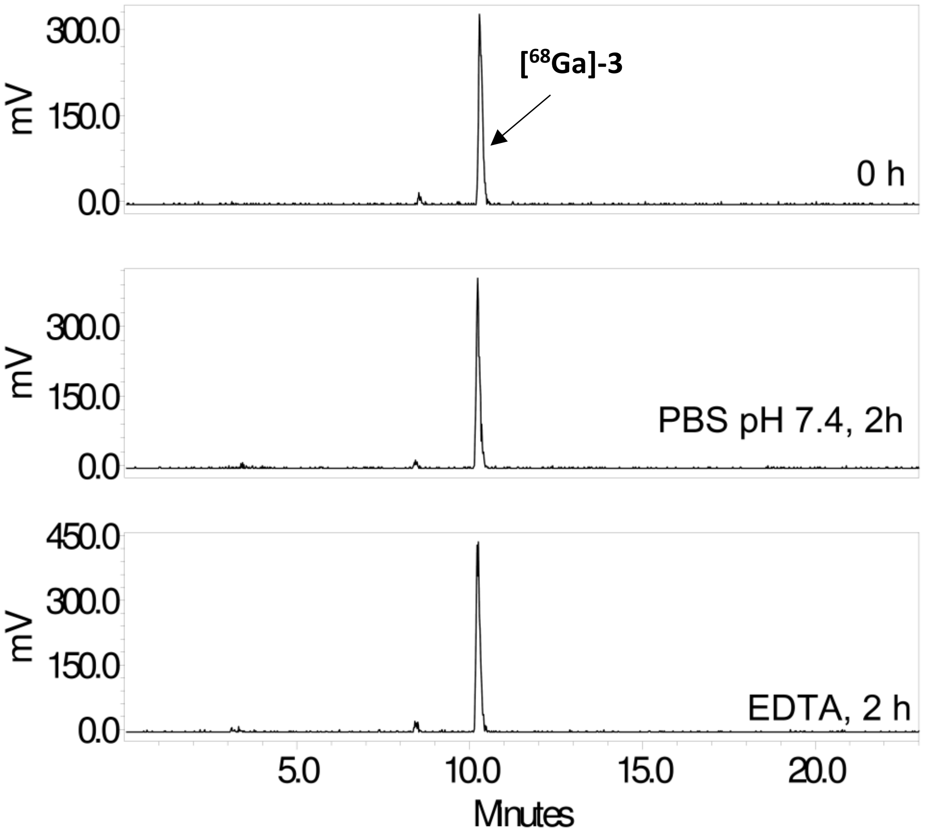

Figure S2. Determination of the radiochemical purity and stability of **[^{68}Ga]-1** (A), **[^{68}Ga]-2** (B) and **[^{68}Ga]-3** (C) by radio-HPLC (system A). The retention time of **[^{68}Ga]-1**, **[^{68}Ga]-2** and **[^{68}Ga]-3** are 10.0, 11.1 and 10.2 min, respectively. The radiochemical purity of **[^{68}Ga]-1**, **2** and **3** were all found to be higher than 98%. The **[^{68}Ga]-1**, **[^{68}Ga]-2** and **[^{68}Ga]-3** remained intact after incubation in PBS (pH 7.4) and EDTA (34 mM) for 2 h.

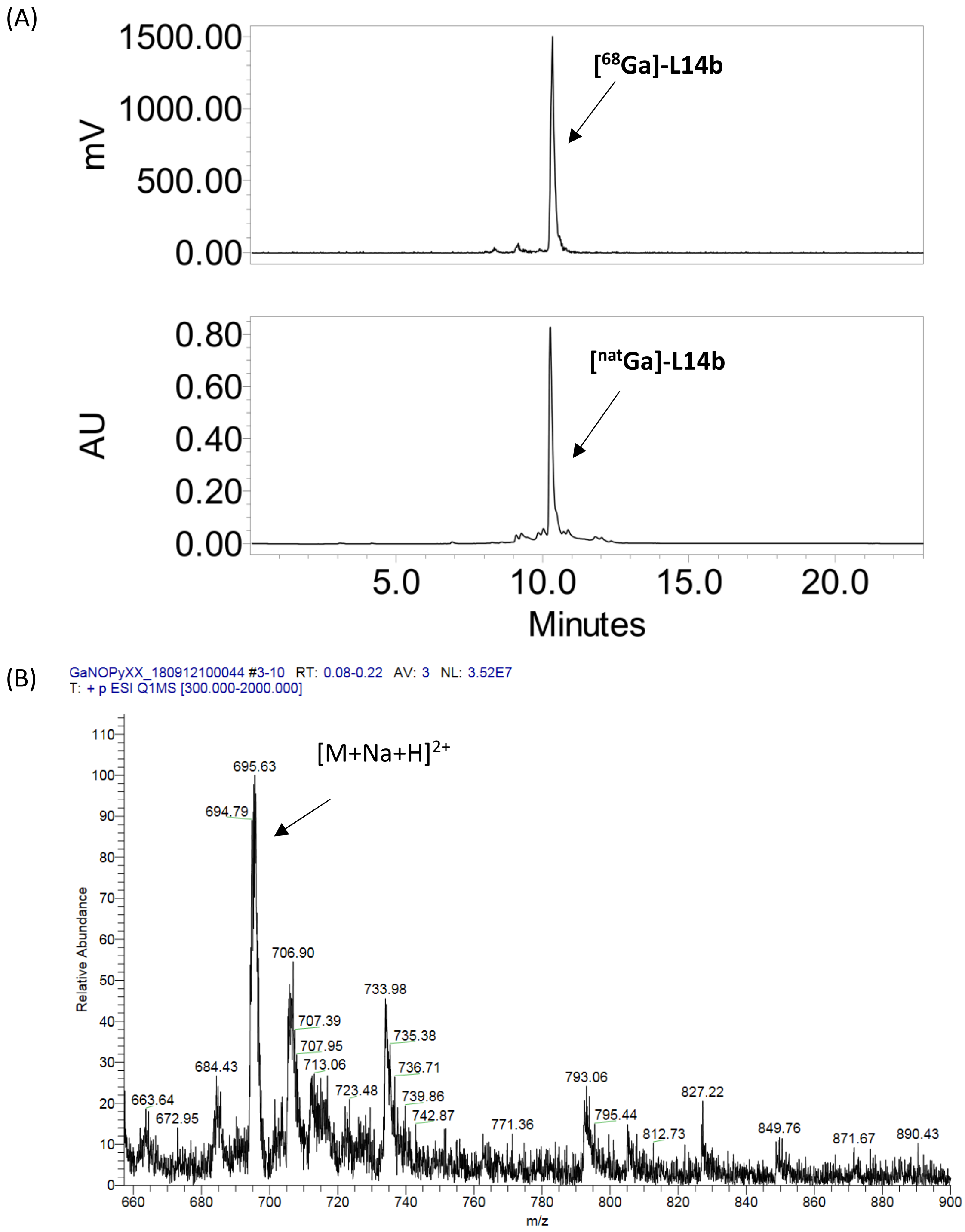

Figure S3. Identification of **[^{68}Ga]-L14b**. (A) Radio-chromatogram of **[^{68}Ga]-L14b** and UV absorbance at 350 nm of its corresponding non-radioactive analog **[^{nat}Ga]-L14b**. The **[^{nat}Ga]-L14b** was prepared following the similar protocol of the preparation of **[^{68}Ga]-L14b**. Briefly, **[^{nat}Ga]-1** (0.8 mg, 1.5 μmol) was labeled by $Ga(NO_3)_3$ (1.5 μmol) in a NaOAc buffer (pH 5.5, 90 ^{o}C, 15 min), and followed by conjugation with **9** (2 mg, 1.5 μmol) in PBS buffer (pH 9.0, 37 ^{o}C, 1 h) to give **[^{nat}Ga]-L14b**. The retention time of **[^{68}Ga]-L14b** and [^{nat}**Ga]-L14b** were 10.3 and 10.3 min, respectively. (B) Mass spectrum of **[^{nat}Ga]-L14b.** ESI-MS: m/z 695.6 $[M + Na + H]^{2+}$.

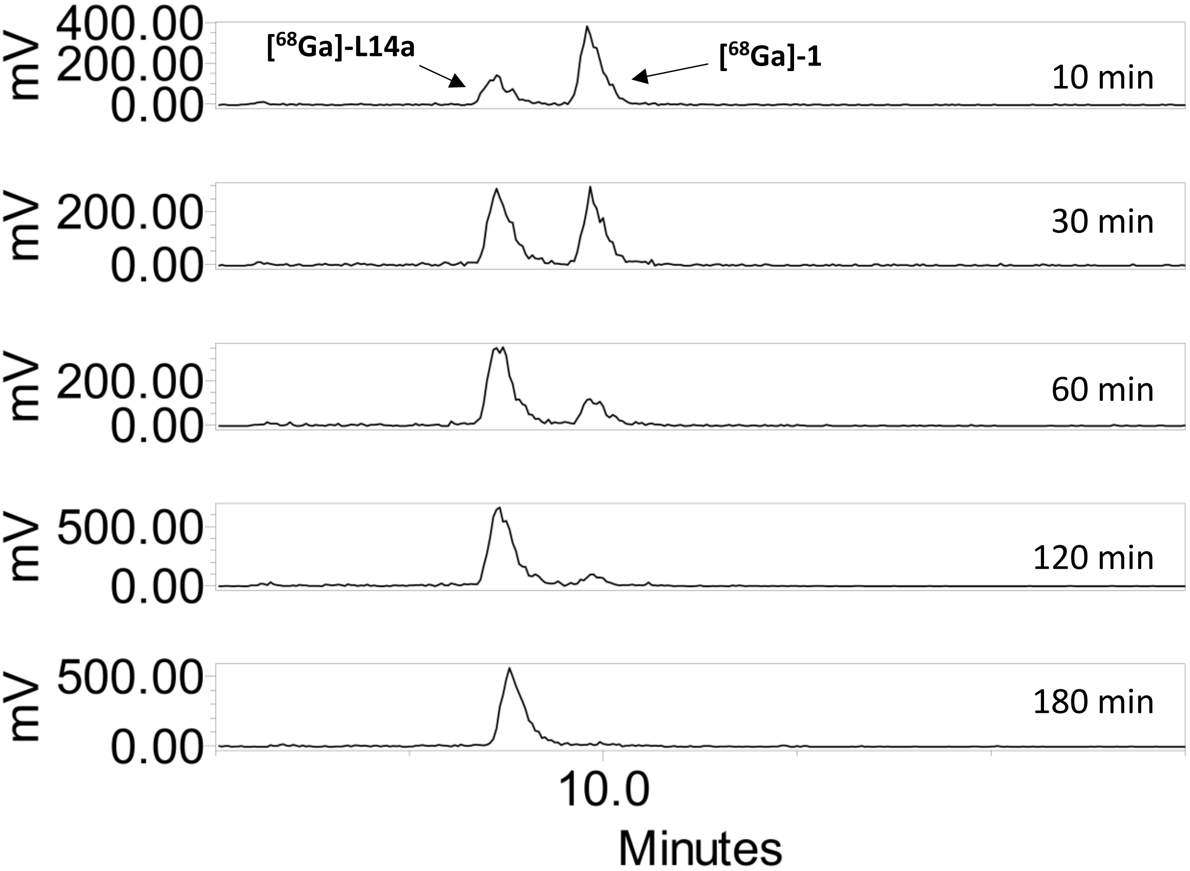

Figure S4. Radio-HPLC monitoring of the progress of click reaction between **[^{68}Ga]-1** and **8**. Conversion of **[^{68}Ga]-1** (retention time = 10.0 min) into the corresponding **[^{68}Ga]-L14a** (retention time = 9.4 min) by treatment with **8** (10 nM) at 37 ^{o}C in PBS (pH 7.4) with agitation (700 rpm). The reactions were quenched by the addition of 10% AcOH and analyzed by analytic radio-HPLC at 10, 30, 60, 120 and 180 min.

(A)

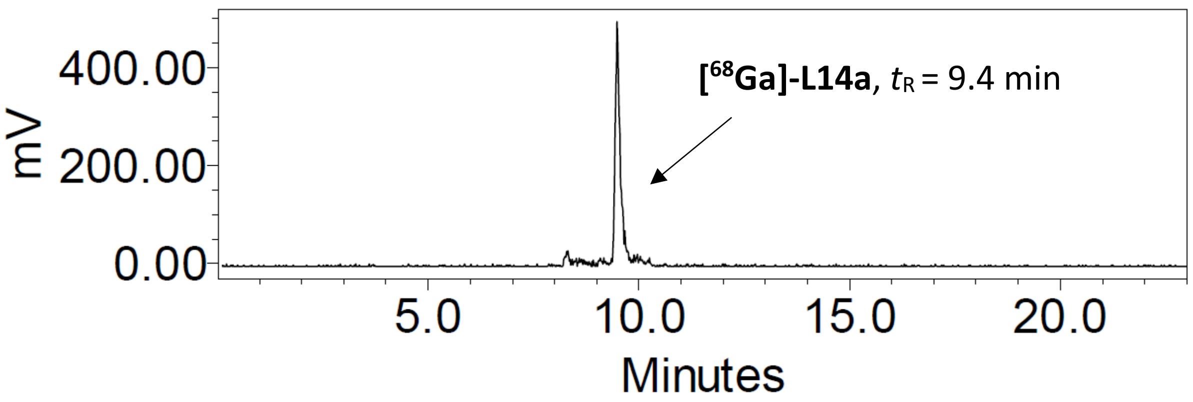

400.00
200.00
0.00
mV
[68Ga]-L14a, tR = 9.4 min
5.0
10.0
15.0
20.0
Minutes

(B)

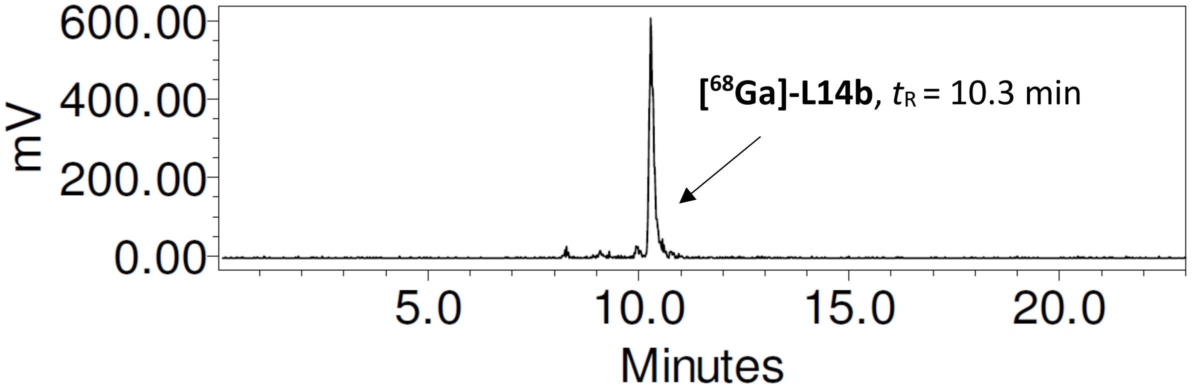

600.00
400.00
200.00
0.00
mV
[68Ga]-L14b, tR = 10.3 min
5.0
10.0
15.0
20.0
Minutes

(C)

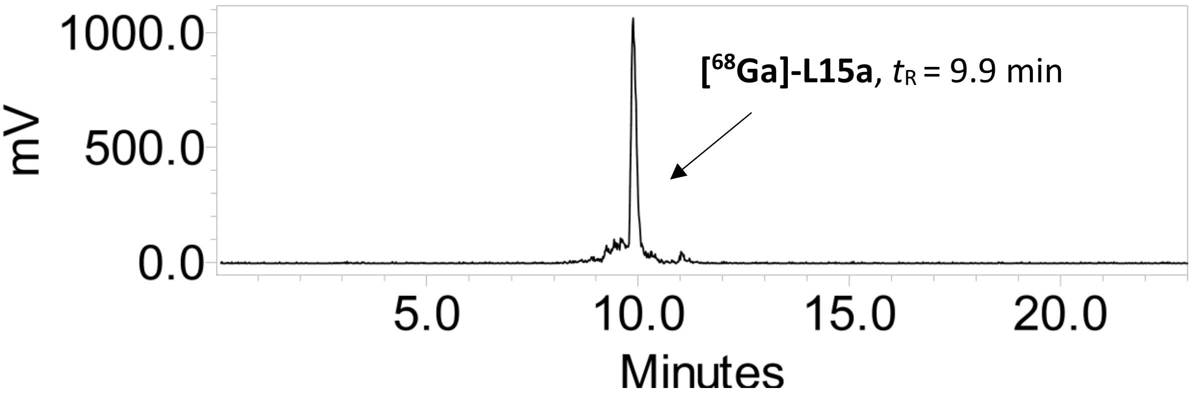

1000.0
500.0
0.0
mV
[68Ga]-L15a, tR = 9.9 min
5.0
10.0
15.0
20.0
Minutes

(D)

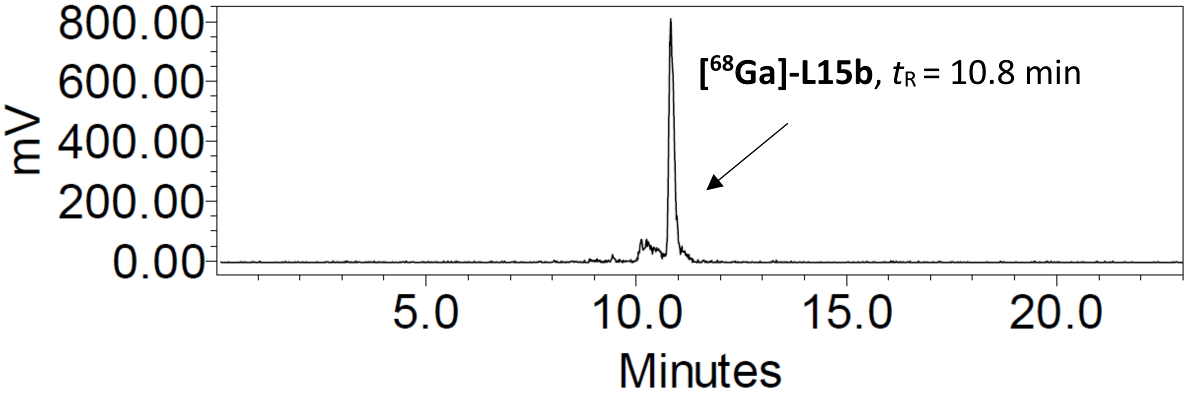

800.00
600.00
400.00
200.00
0.00
mV
[68Ga]-L15b, tR = 10.8 min
5.0
10.0
15.0
20.0
Minutes

(E)

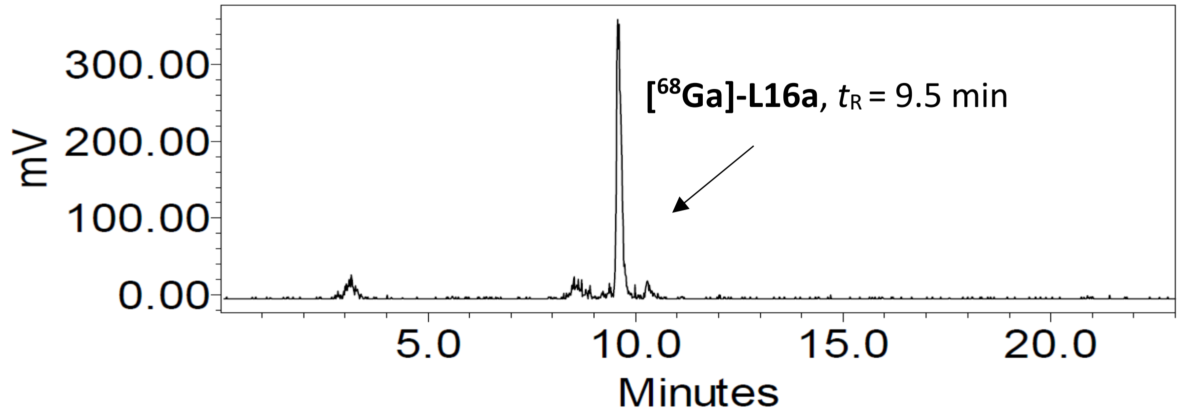

(F)

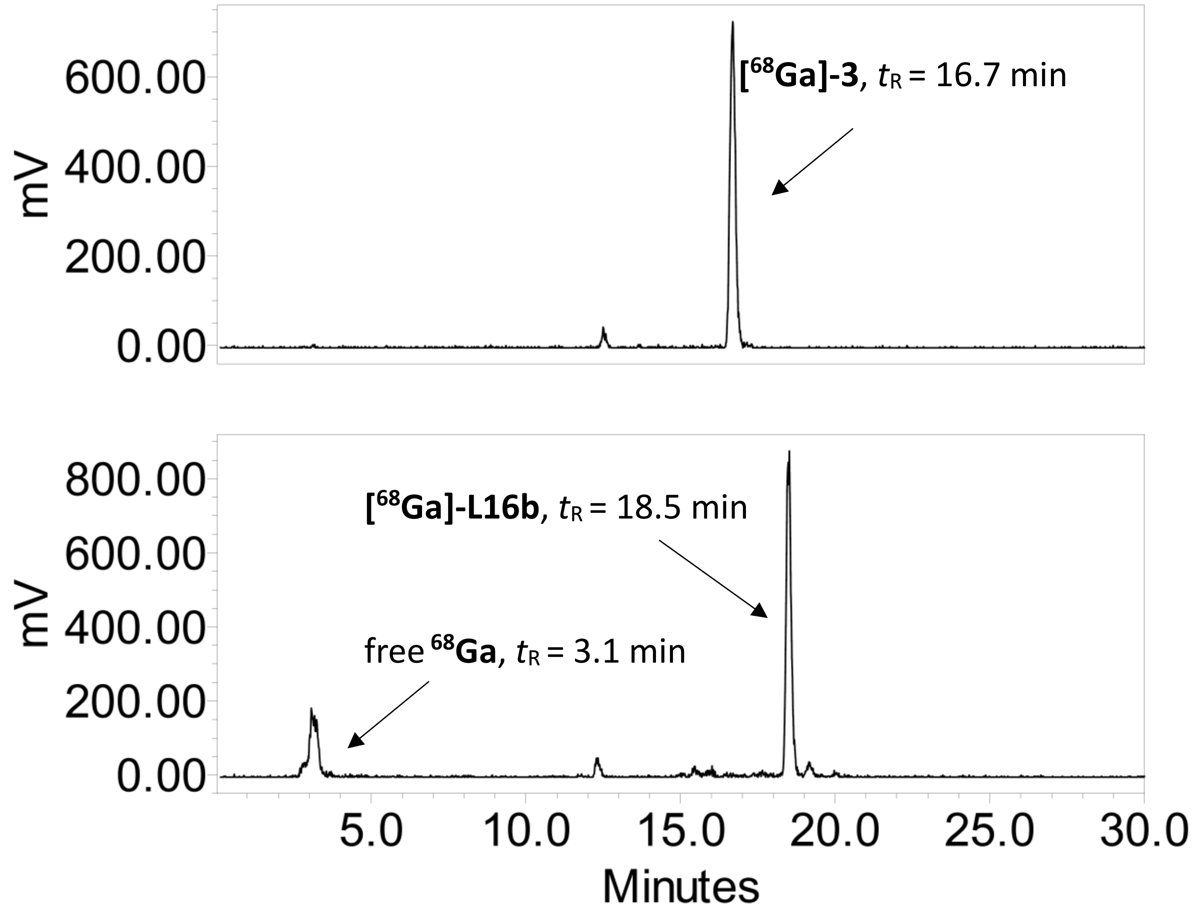

Figure S5. Radio-HPLC monitoring of the click reaction between **[^{68}Ga]**-CBTs and AAZs. Syntheses of **[^{68}Ga]-L14a** (A), **[^{68}Ga]-L14b** (B), **[^{68}Ga]-L15a** (C), **[^{68}Ga]-L15b** (D) and **[^{68}Ga]-L16a** (E) were analyzed by HPLC system A. The preparation of **[^{68}Ga]-L16b** (F: bottom) was analyzed by HPLC system B. The retention time of the precursor **[^{68}Ga]-3** (F: top) was indicated as 16.7 min in this system.

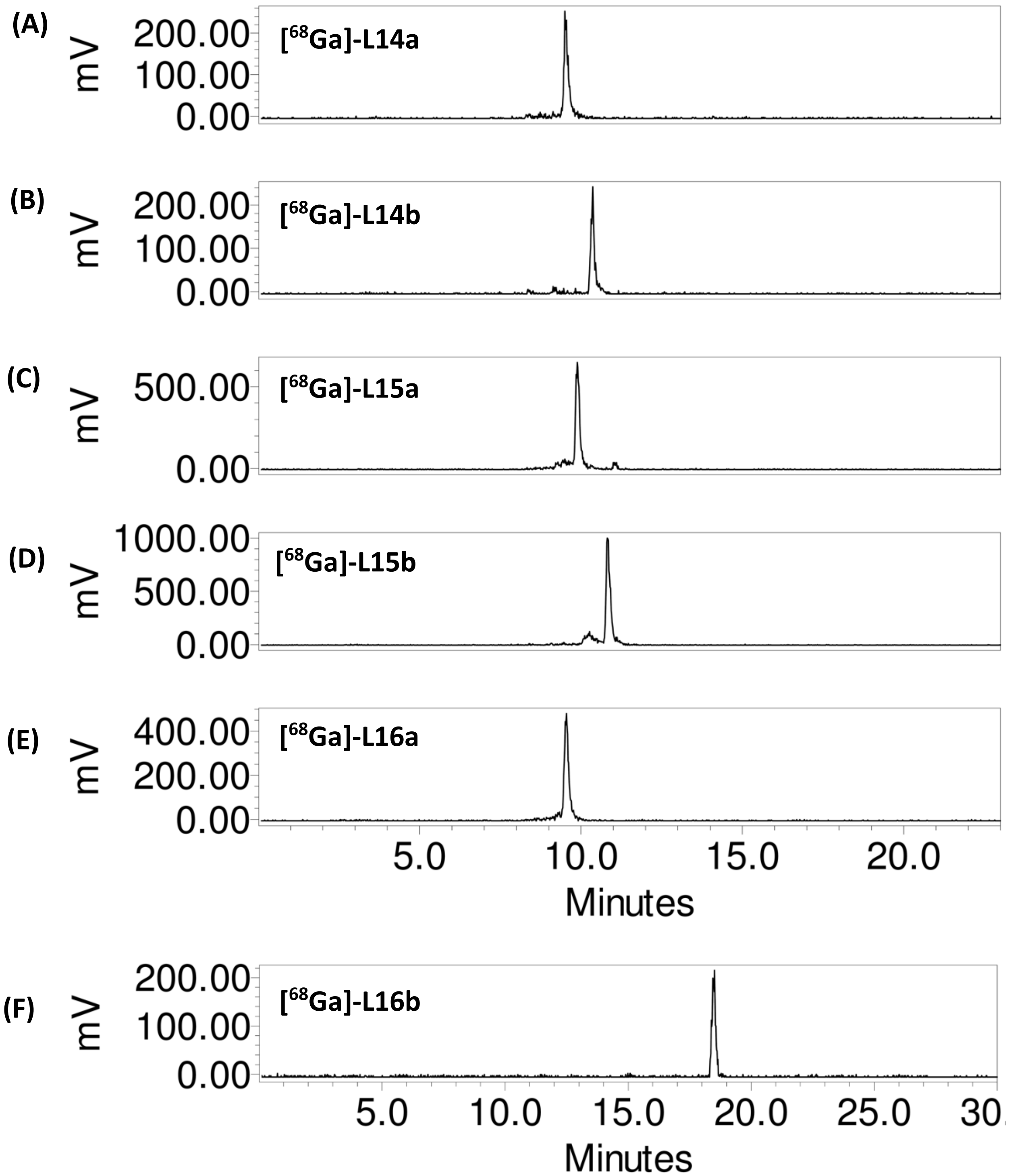

Figure S6. Determination of the stability of **[^{68}Ga]-L14**-L**16** in PBS by radio-HPLC. The stability of **[^{68}Ga]-L14a** (A), **[^{68}Ga]-L14b** (B), **[^{68}Ga]-L15a** (C), **[^{68}Ga]-L15b** (D) and **[^{68}Ga]-L16a** (E) were analyzed by HPLC system A, and **[^{68}Ga]-L16b** (F) was analyzed by HPLC system B. **[^{68}Ga]-L16a** and **[^{68}Ga]-L16b** were purified by HPLC to remove free ^{68}Ga before stability testing. All the compounds were stable after incubation in PBS (pH 7.4) for 2 h, and no radiolysis were observed.

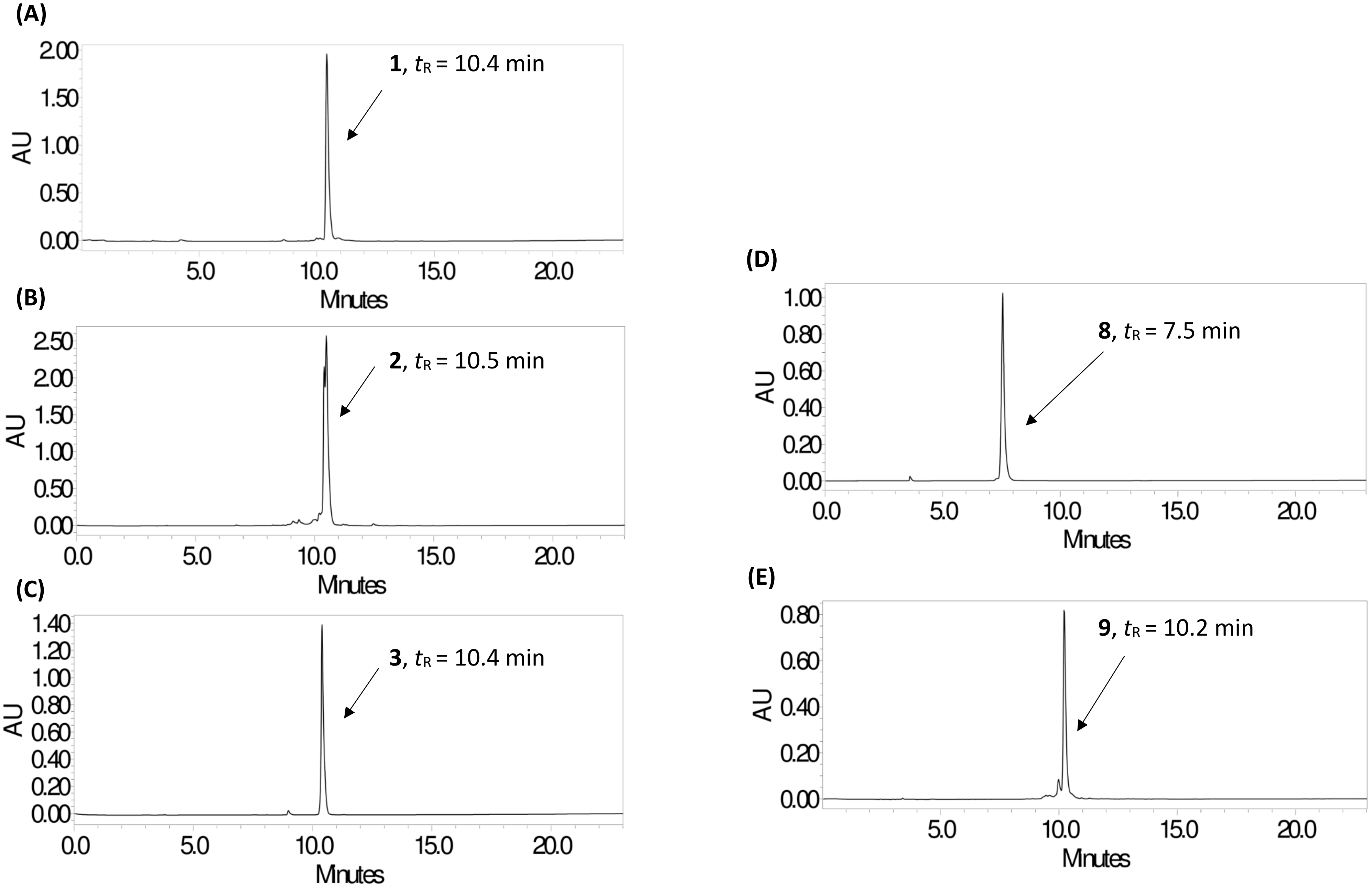

Figure S7. Determination of the purity of **1** (A), **2** (B), **3** (C), **8** (D) and **9** (E) by HPLC (system A, UV detector at 254 nm). The retention time of **1**, **2**, **3**, **8** and **9** are 10.4, 10.5, 10.4, 7.5 and 10.2 min, respectively. The purity of **1**, **2**, **3**, **8** and **9** were all found to be higher than 95%.

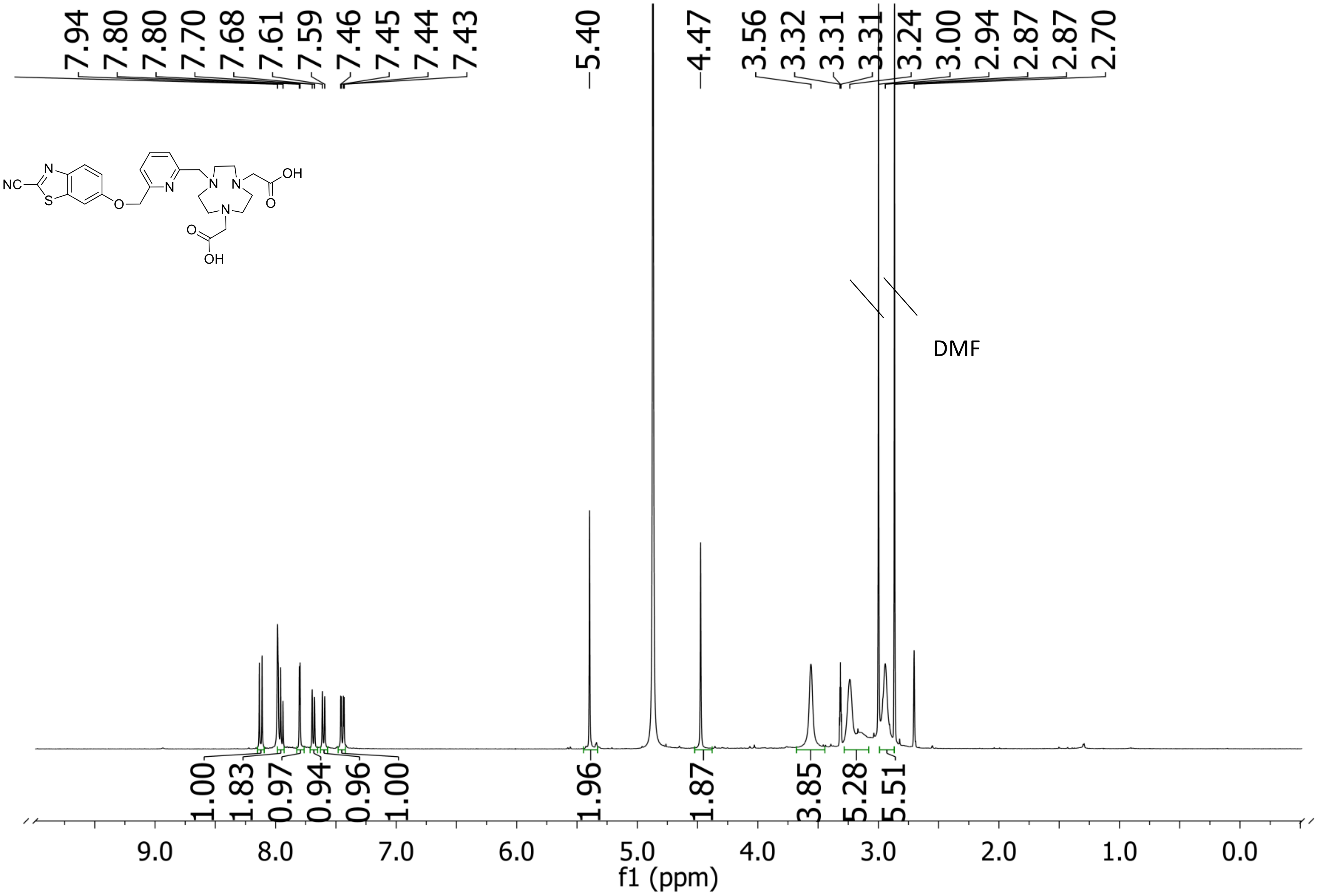

Figure S8. ^{1}H-NMR Spectra of compound **1** (400 MHz, CD_3OD)

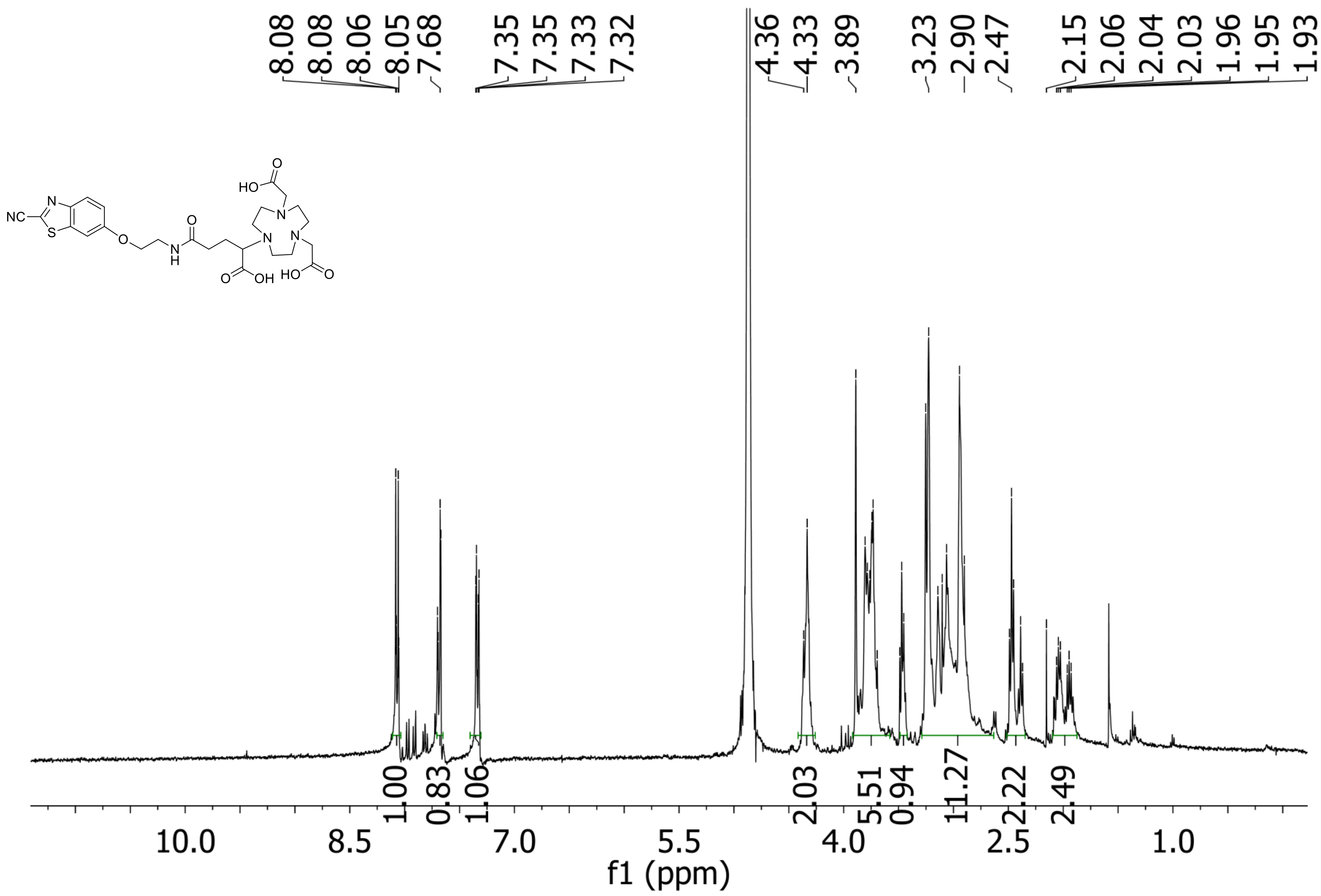

Figure S9. ^{1}H-NMR Spectra of compound **2** (400 MHz, CD_3OD).

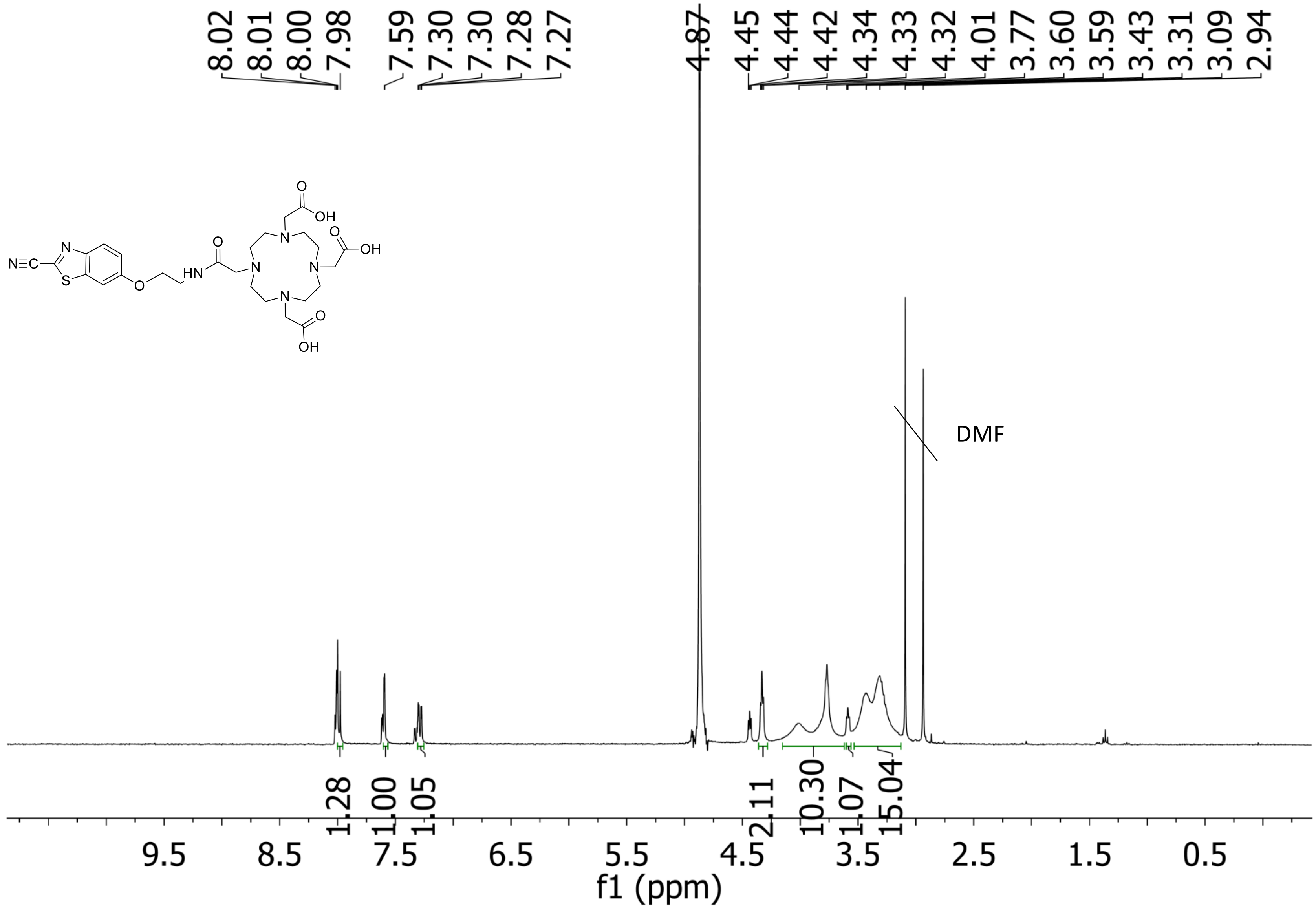

Figure S10. ^{1}H-NMR Spectra of compound **3** (400 MHz, CD_3OD).

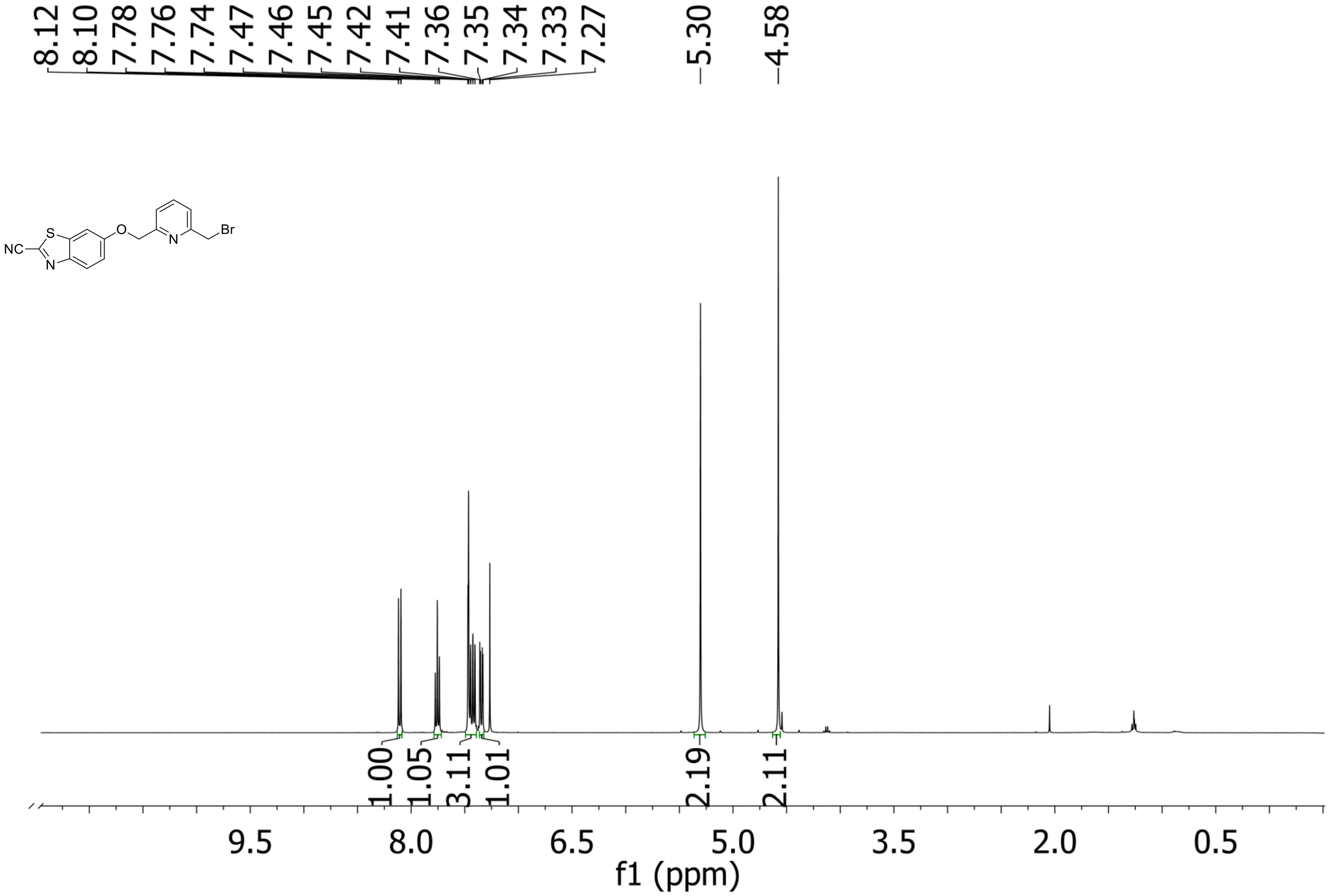

Figure S11. ^{1}H-NMR Spectra of compound **5** (400 MHz, $CDCl_3$).

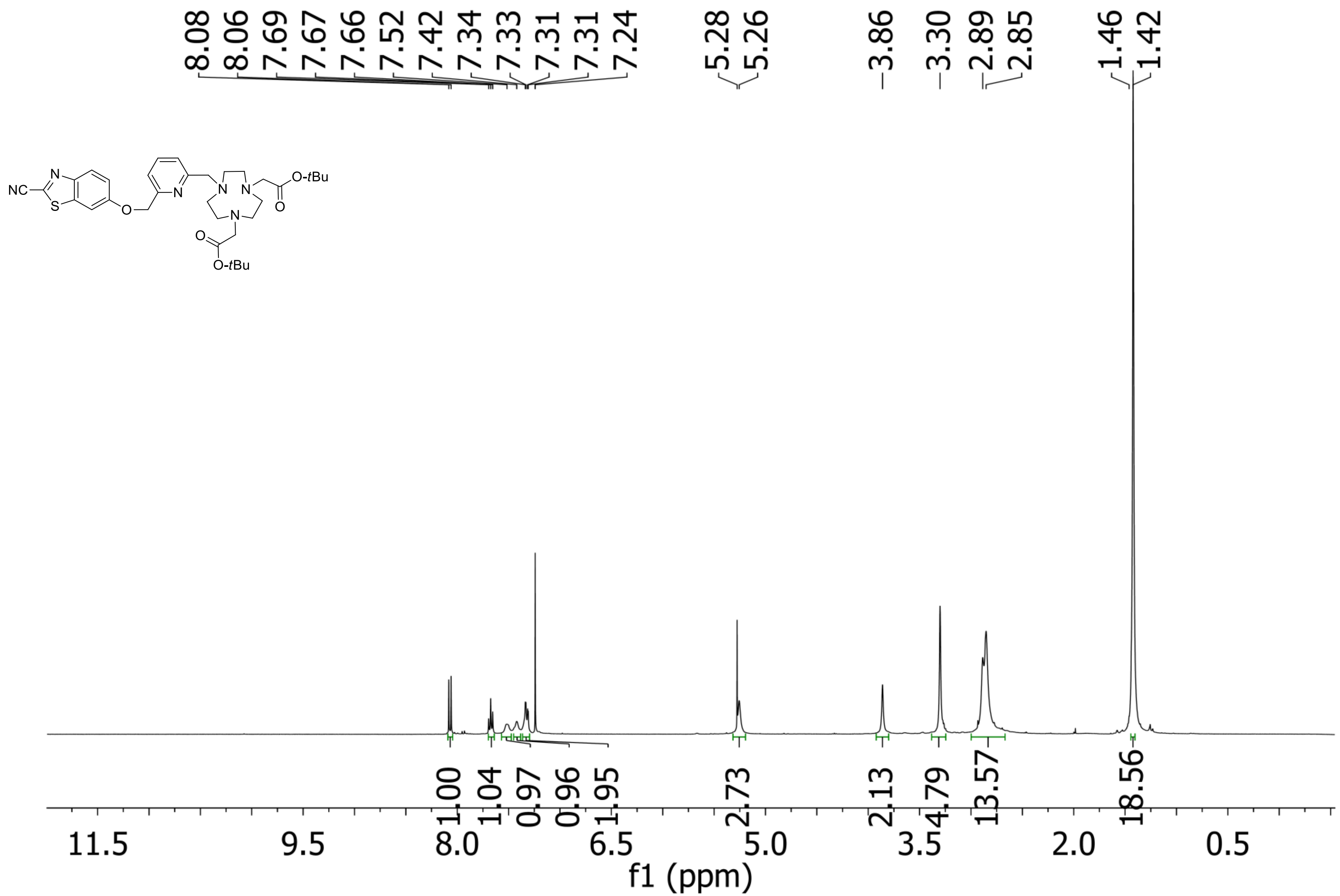

Figure S12. ^{1}H-NMR Spectra of compound **6** (400 MHz, $CDCl_3$).

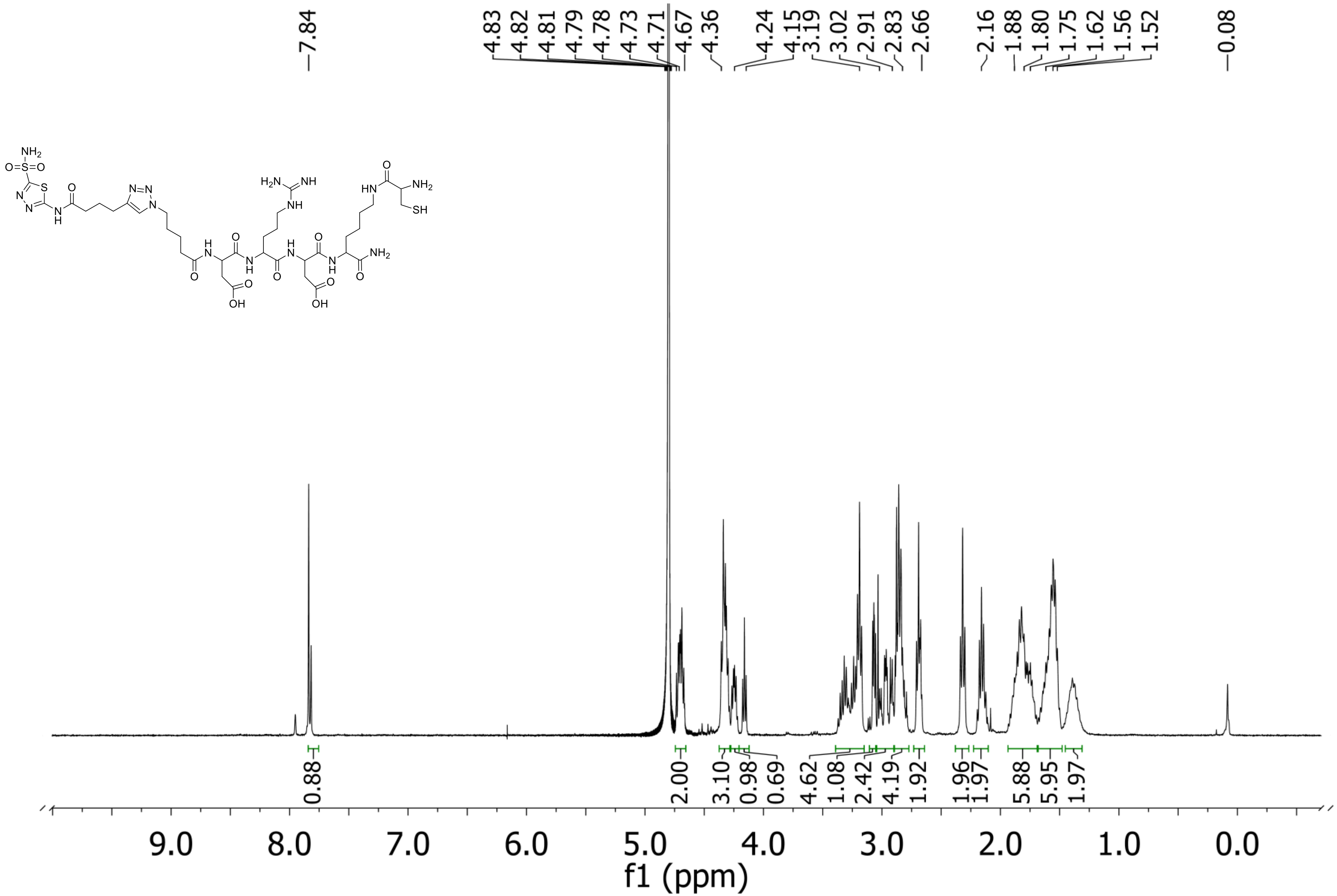

Figure S13. ^{1}H-NMR Spectra of compound **8** (400 MHz, D_2O).

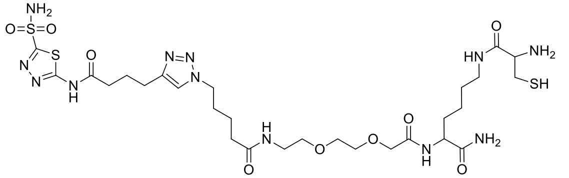

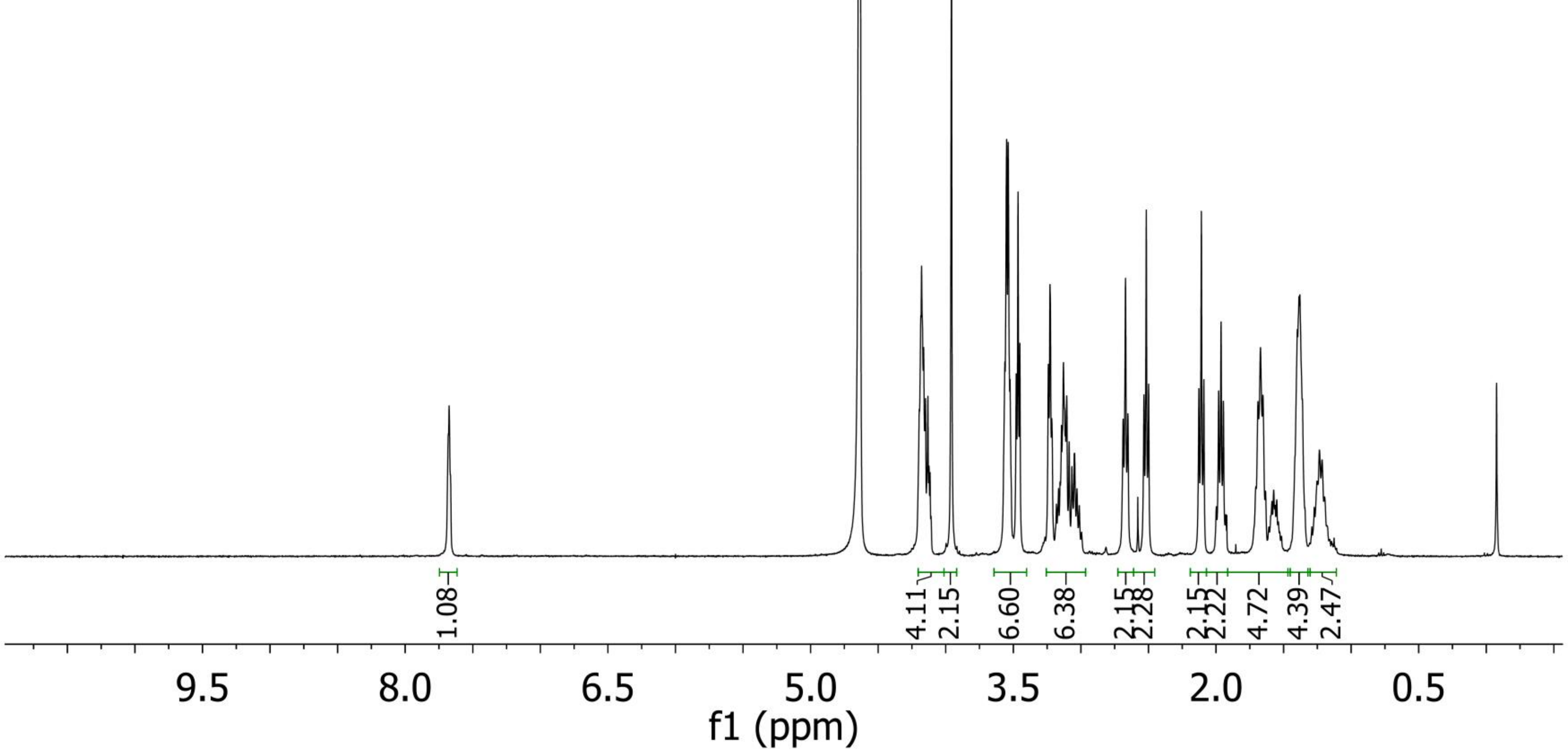

Figure S14. ^{1}H-NMR Spectra of compound **9** (400 MHz, D_2O).

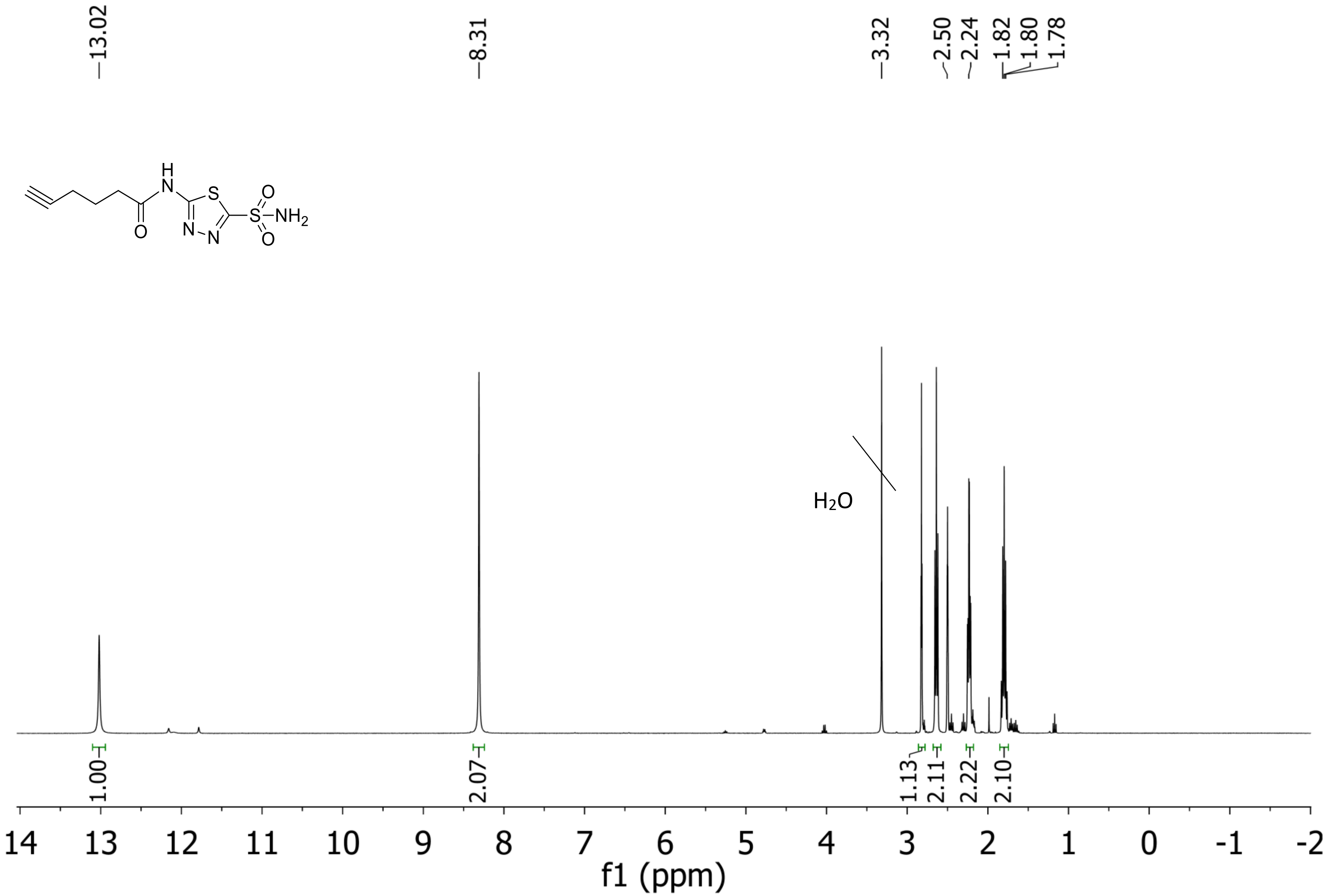

Figure S15. ^{1}H-NMR Spectra of compound **13** (400 MHz, DMSO-*d*6).
